# Cholinergic macrophages promote the resolution of peritoneal inflammation

**DOI:** 10.1073/pnas.2402143121

**Published:** 2024-06-26

**Authors:** Shufeng Luo, Huiling Lin, Chong Wu, Lan Zhu, Qiaomin Hua, Yulan Weng, Lu Wang, Xiaoli Fan, Kai-Bo Zhao, Gaoteng Liu, Yuting Wang, Hai-Tian Chen, Li Xu, Limin Zheng

**Affiliations:** ^a^State Key Laboratory of Oncology in South China, Guangdong Provincial Clinical Research Center for Cancer, Sun Yat-sen University Cancer Center, Sun Yat-sen University, Guangzhou 510060, People’s Republic of China; ^b^Guangdong Provincial Key Laboratory of Pharmaceutical Functional Genes, Ministry of Education Key Laboratory of Gene Function and Regulation, School of Life Sciences, Sun Yat-sen University, Guangzhou 510275, People’s Republic of China; ^c^Department of Obstetrics and Gynecology, The First Affiliated Hospital of Sun Yat-sen University, Guangzhou 510080, People’s Republic of China

**Keywords:** acetylcholine, macrophages, inflammation, peritoneal cavity

## Abstract

The non-neural cholinergic system plays a critical role in regulating immune equilibrium and tissue homeostasis. While the expression of choline acetyltransferase (ChAT), the enzyme catalyzing acetylcholine biosynthesis, has been well documented in lymphocytes, its role in the myeloid compartment is less understood. Here, we identify a significant population of macrophages (Mϕs) expressing ChAT and synthesizing acetylcholine in the resolution phase of acute peritonitis. Using *Chat*-GFP reporter mice, we observed marked upregulation of ChAT in monocyte-derived small peritoneal Mϕs (SmPMs) in response to Toll-like receptor agonists and bacterial infections. These SmPMs, phenotypically and transcriptionally distinct from tissue-resident large peritoneal macrophages, up-regulated ChAT expression through a MyD88-dependent pathway involving MAPK signaling. Notably, this process was attenuated by the TRIF-dependent TLR signaling pathway, and our tests with a range of neurotransmitters and cytokines failed to induce a similar response. Functionally, *Chat* deficiency in Mϕs led to significantly decreased peritoneal acetylcholine levels, reduced efferocytosis of apoptotic neutrophils, and a delayed resolution of peritonitis, which were reversible with exogenous ACh supplementation. Intriguingly, despite B lymphocytes being a notable ChAT-expressing population within the peritoneal cavity, *Chat* deletion in B cells did not significantly alter the resolution process. Collectively, these findings underscore the crucial role of Mϕ-derived acetylcholine in the resolution of inflammation and highlight the importance of the non-neuronal cholinergic system in immune regulation.

## Significance

Acetylcholine, originally recognized as a neurotransmitter, is increasingly acknowledged as a key immune modulator in various physiological and pathological conditions. This study unveils a population of cholinergic macrophages (Mϕs), which are crucial in resolving acute peritonitis. These Mϕs, expressing the enzyme choline acetyltransferase (ChAT) and synthesizing acetylcholine, are primarily monocyte-derived small peritoneal Mϕs, distinct from tissue-resident large peritoneal Mϕs. The selective deficiency of ChAT in Mϕs impedes efferocytosis and delays apoptotic neutrophils clearance, effects reversible by acetylcholine supplementation. These findings illuminate the pivotal role of Mϕ-mediated cholinergic regulation in inflammation resolution and enhance our understanding of the non-neuronal cholinergic system’s role in immune regulation.

Acute inflammation is a fundamental and dynamic biological response. In addition to its crucial role in defending the host against microbial invasion or tissue injury, this process is also characterized by a highly regulated and self-limiting nature. Once the immunological threat is resolved, the immune system activates intricate mechanisms to prevent the inflammation from transitioning into a prolonged and chronic state (1–5). Macrophages (Mϕs) play a pivotal role in initiating, promoting, and resolving inflammation. In the resolution phase, these cells clear the dead cells through efferocytosis and produce anti-inflammatory mediators that are integral for tissue repair and restoring microenvironmental homeostasis (6–10). However, the molecular mechanisms that govern these Mϕ functions during the transition from active inflammation to resolution have not been fully elucidated. An in-depth exploration of Mϕ ontogeny and functional attributes in this context is essential for understanding their roles and potential mechanisms in modulating the inflammatory process.

Acetylcholine (ACh), generally known for its role as a neurotransmitter in muscle contraction, neuron communication, and vasodilation, has been increasingly recognized for its effect on non-neuronal systems (11–14). Various immune cells, including T cells, B cells, NK cells, and adipose-resident Mϕs, have been shown to conditionally express choline acetyltransferase (ChAT), the enzyme that catalyzes the rate-limiting step of ACh production (15–20). These recent findings position ChAT-expressing immune cells at the intersection of immune and cholinergic signaling. Intriguingly, immune cell–derived ACh plays context-dependent roles in immune regulation, enhancing antiviral responses in lymphocytic choriomeningitis virus (LCMV) infection (21) while inhibiting proinflammatory cytokine production in other conditions, such as sepsis (15). This complexity underscores the importance of understanding the distinct roles and dynamics of ChAT-expressing immune cells in various pathological states.

In this study, we identify a critical role of Mϕs as cholinergic immune cells in resolving acute inflammation within the peritoneal cavity. These Mϕs, primarily a subset of small peritoneal Mϕs (SmPMs) of monocyte origin, up-regulated ChAT expression via Toll-like receptor (TLR)-MyD88 signaling. Notably, this process was attenuated by the TRIF-dependent TLR signaling pathway. Selective deletion of *Chat* in Mϕs impeded the resolution of inflammation, a phenomenon that was reversed by ACh supplementation. These findings uncover a unique aspect of the non-neuronal cholinergic axis in the myeloid compartment, highlighting its critical role in controlling the resolution of inflammation.

## Results

### Emergence of ChAT-Expressing Mϕs during the Resolution Phase of Acute Peritonitis.

**Fig. 1.**
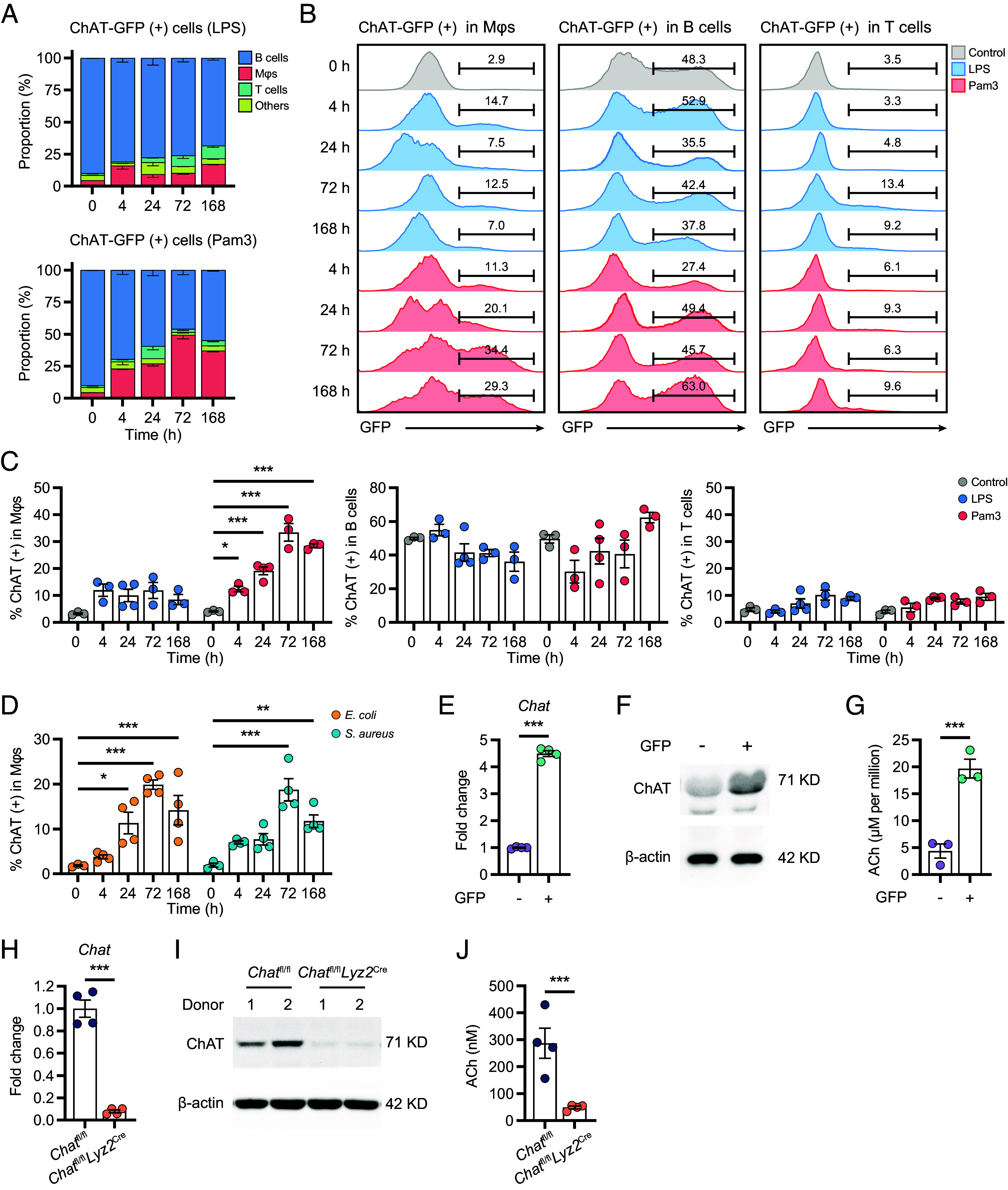
Upregulation of ChAT expression in Mϕs during the resolution of inflammation. (*A*) Constitution of the ChAT-expressing (GFP^+^) population in peritoneal lavages at the indicated time points after intraperitoneal administration of either saline (indicated as 0 h), LPS, or Pam3. (*B* and *C*) Representative histogram (*B*) and percentages (*C*) of ChAT-expressing cells in Mϕs, B, and T cells. (*D*) Mice were inoculated with 10^6^ CFU of either *E. coli* or *S. aureus* by intraperitoneal injection, and percentages of ChAT-expressing cells in Mϕs were analyzed by flow cytometry. (*E* and *F*) ChAT expression within GFP^−^ and GFP^+^ peritoneal Mϕs was assessed via qPCR (*E*) and immunoblotting (*F*). (*G*) ACh production was assessed by UPLC–MS/MS in GFP^−^ and GFP^+^ peritoneal Mϕs isolated from Pam3-treated (72 h) ChAT-GFP mice. (*H* and *I*) ChAT expression in Pam3-treated (72 h) peritoneal Mϕs from *Chat*^fl/fl^ and *Chat*^fl/fl^*Lyz2*^cre^ mice was determined by qPCR (*H*) and immunoblotting (*I*). (*J*) The concentration of ACh in peritoneal lavage fluid from Pam3-treated (72 h) *Chat*^fl/fl^ and *Chat*^fl/fl^*Lyz2*^cre^ mice was determined by UPLC–MS/MS. Data are from three independent experiments. Error bars indicate mean ± SEM. Statistics: (*C* and *D*) Two-way ANOVA corrected by Šidák’s test. (*E*, *G*, *H*, and *J*) Student’s *t* test. *P < 0.05, **P < 0.01, and ***P < 0.001. *E. coli*, *Escherichia coli*; *S. aureus*, *Staphylococcus aureus.*

ChAT expression plays multifaceted roles in regulating immune responses within the immune system (15–17). These recent findings highlight the need to delineate and clarify the heterogeneous population of ChAT-expressing immune cells and their functional dynamics within specific pathological contexts. To this end, we analyzed the expression pattern of ChAT in the peritoneal lavage of reporter mice that express enhanced green fluorescent protein (GFP) under the control of transcriptional regulatory elements of the *Chat* gene (*Chat*-GFP reporter mice) (22). Under resting conditions, B cells constituted the dominant GFP-positive population in peritoneal lavage (Fig. 1*A*, labeled “0 h”). A similar pattern was observed in lipopolysaccharide (LPS; a TLR4 agonist)-induced sterile peritonitis (Fig. 1 *A*, *Upper*), albeit with a slight increase in GFP expression among Mϕs and T cells (15% in Mϕs and 10% in T cells at their peak; Fig. 1 *A*, *Upper*). In contrast, we observed a striking time-dependent increase in GFP-expressing Mϕs in response to Pam3CSK4 (Pam3), a TLR1/2 agonist (Fig. 1 *A*, *Lower*). Following Pam3 stimulation, GFP expression in Mϕs was evident as early as 4 h poststimulation, corresponding to the peak phase of leukocyte recruitment in acute peritonitis (*SI Appendix*, Fig. S1*A*). By day 3, Mϕs constituted approximately 50% of the GFP^+^ cells (Fig. 1 *A*, *Lower*), with more than 30% of the intraperitoneal Mϕs exhibiting GFP expression (Fig. 1 *B* and *C*). At this time point, the inflammatory response was in the resolution phase, as evidenced by the significant decrease in neutrophils and monocytes (*SI Appendix*, Fig. S1*A*). Meanwhile, the elevation of GFP expression in T cells, B cells, and neutrophils was only marginal (Fig. 1 *B* and *C* and *SI Appendix*, Fig. S1*B*). The Pam3-induced *Chat*-GFP expression in resolution-phase Mϕs was consistent in experiments using various administration doses (*SI Appendix*, Fig. S1*C*). In comparison, even though LPS could induce similar peritonitis at the acute phase (*SI Appendix*, Fig. S1*D*), its impact on *Chat*-GFP expression in resolution-phase Mϕs was mild regardless of administration doses (*SI Appendix*, Fig. S1*C*). To further evaluate the expression of ChAT in Mϕs during bacterial infection, we adopted a self-resolving model of peritonitis induced by *Escherichia coli* or *Staphylococcus aureus* (23). The expression of ChAT Mϕs was significantly increased at the resolution phase (72 h) but not at the acute phase of either *E. coli*-induced peritonitis (4 h) or *S. aureus*-induced peritonitis (24 h) (Fig. 1*D* and *SI Appendix*, Fig. S1*E*).

To corroborate the results obtained with the *Chat*-GFP reporter system, we sorted both GFP-negative and GFP-positive peritoneal Mϕs (*SI Appendix*, Fig. S1*F*) and validated the Pam3-induced expression of ChAT at the mRNA (Fig. 1*E*) and protein (Fig. 1*F*) levels in these cells. Furthermore, we detected an average intracellular ACh level of ~20 µM per million *Chat*-GFP-positive peritoneal Mϕs, remarkably higher than that in their GFP-negative counterparts (Fig. 1*G*). Genetic deletion of the fourth and fifth exons of *Chat*, guided by *Lyz2*^cre^ (*Chat*^fl/fl^*Lyz2*^cre^ mice), led to an almost complete loss of ChAT protein expression in Pam3-stimulated peritoneal Mϕs (Fig. 1 *H* and *I*). In addition, we found an over 80% ACh reduction in the peritoneal lavage from Pam3-treated *Chat*^fl/fl^*Lyz2*^cre^ mice compared to *Chat*^fl/fl^ mice (Fig. 1*J*), consistent with the findings that Mϕs were the main ChAT-expression cell population at this time point and the high intracellular ACh level in these cells. Collectively, our findings underscore the significant upregulation of ChAT and ACh synthesis in peritoneal Mϕs during the resolution phase of the Pam3-induced inflammatory response.

### Phenotypic Characteristics of ChAT-Expressing Peritoneal Mϕs.

**Fig. 2.**
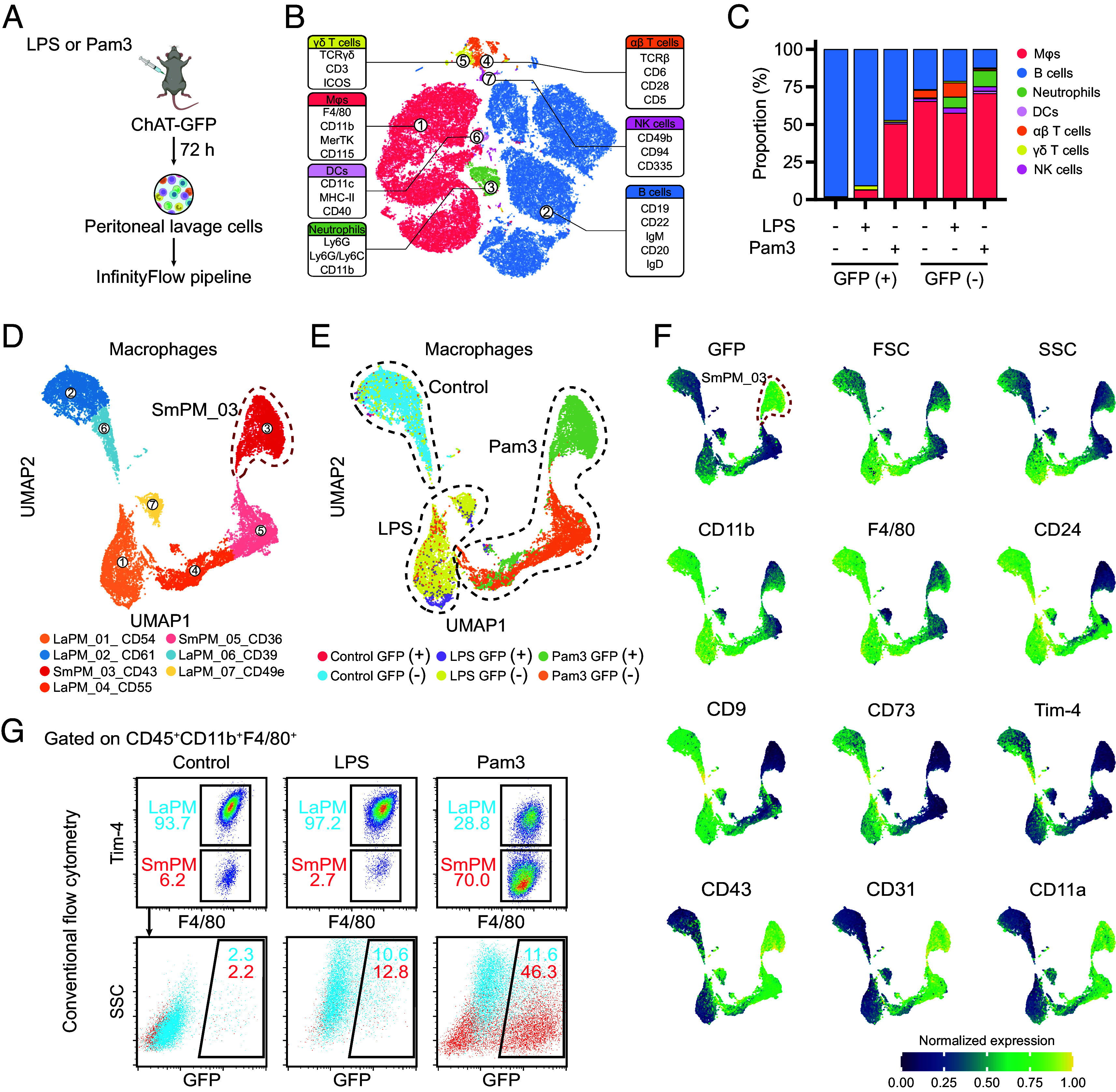
InfinityFlow analysis reveals the ChAT enrichment in F4/80^lo^Tim-4^−^ small peritoneal Mϕs. (*A*) Scheme of the InfinityFlow analysis on the cell surface markers. (*B*) Cellular discrimination was achieved through t-SNE dimensionality reduction, with clustering via FlowSOM and ConsensusClusterPlus based on the 93 discriminating markers defined in *SI Appendix*, Table S1. (*C*) The frequencies of GFP^−^ and GFP^+^ peritoneal leukocytes at 72 h posttreatment. (*D*) Subclustering of peritoneal Mϕs based on uniform manifold approximation and projection (UMAP), revealing distinct phenotypic cluster profiles detailed in Dataset S1. (*E*) UMAP of peritoneal Mϕs from control, LPS-, or Pam3-treated mice. (*F*) Normalized expression levels of various markers on peritoneal Mϕs, determined by InfinityFlow. (*G*) Percentages of F4/80^hi^Tim-4^+^ LaPMs and F4/80^lo^Tim-4^−^ SmPMs (*Upper*) and the proportions of ChAT expression in these cells (*Lower*) were analyzed by conventional flow cytometry in control, LPS-, or Pam3-treated mice. Representative results of three independent experiments are shown.

To delineate the phenotypic characteristics of ChAT-expressing peritoneal immune cells, we performed InfinityFlow analysis, a multiparametric flow cytometry approach (24, 25), to profile the expression patterns of 253 surface protein markers under various conditions (Fig. 2*A*). Seven primary peritoneal immune subtypes were identified: Mϕs, neutrophils, B cells, dendritic cells (DCs), natural killer (NK) cells, αβ T cells, and γδ T cells (Fig. 2*B* and *SI Appendix*, Fig. S2*A* and Table S1). These cell types could be further classified into 23 specific subpopulations based on unique phenotypic profiles (Fig. 2 *B*–*D* and *SI Appendix*, Fig. S2*B* and Dataset S1). Notably, LPS and Pam3 not only exhibited different capacities to induce ChAT expression in Mϕs at various doses (Figs. 1 *A* and *B* and 2*C* and *SI Appendix*, Fig. S1*C*), but rather activated differential Mϕ responses, characterized by the emergence of different Mϕ clusters (Fig. 2 *D* and *E*).

The peritoneal cavity hosts two distinct Mϕ subsets: tissue-resident large peritoneal Mϕs (LaPMs) and smaller, monocyte-derived Mϕs (MDMs) termed small peritoneal Mϕs (SmPMs) (26, 27). In contrast to homeostatic and LPS-induced conditions, Pam3 stimulation led to a significant accumulation of SmPMs, characterized by a smaller size and lower granularity, as indicated by reduced forward scatter (FSC) and side scatter (SSC) signals, respectively (26) (Fig. 2 *E* and *F*). Moreover, these Mϕs exhibited lower expression levels of typical LaPM markers such as CD11b, F4/80, CD24, CD9, CD73, and Tim-4 (28) and displayed increased expression of the adhesion molecules CD43, CD31, and CD11a. These characteristics of SmPMs were even more pronounced in the Pam3-induced GFP-positive Mϕs (Fig. 2 *E* and *F*), suggesting selectively enriched GFP expression in SmPMs. Conventional flow cytometry analysis confirmed that over 70% of peritoneal Mϕs at 72 h after various doses of Pam3 stimulation were classified as F4/80^lo^Tim-4^−^ SmPMs, whereas less than 10% of these cells were found in homeostatic and LPS-treated conditions (Fig. 2*G* and *SI Appendix*, Fig. S2*C*). Furthermore, in these Pam3-induced SmPMs, more than 40% of the cells were GFP positive, in contrast to the low levels (≤15%) of GFP expression in both LaPMs and SmPMs under homeostatic and LPS-stimulated conditions (Fig. 2*G*).

### MyD88-MAPK p38/ERK Signaling Mediates TLR-Driven ChAT Expression.

**Fig. 3.**
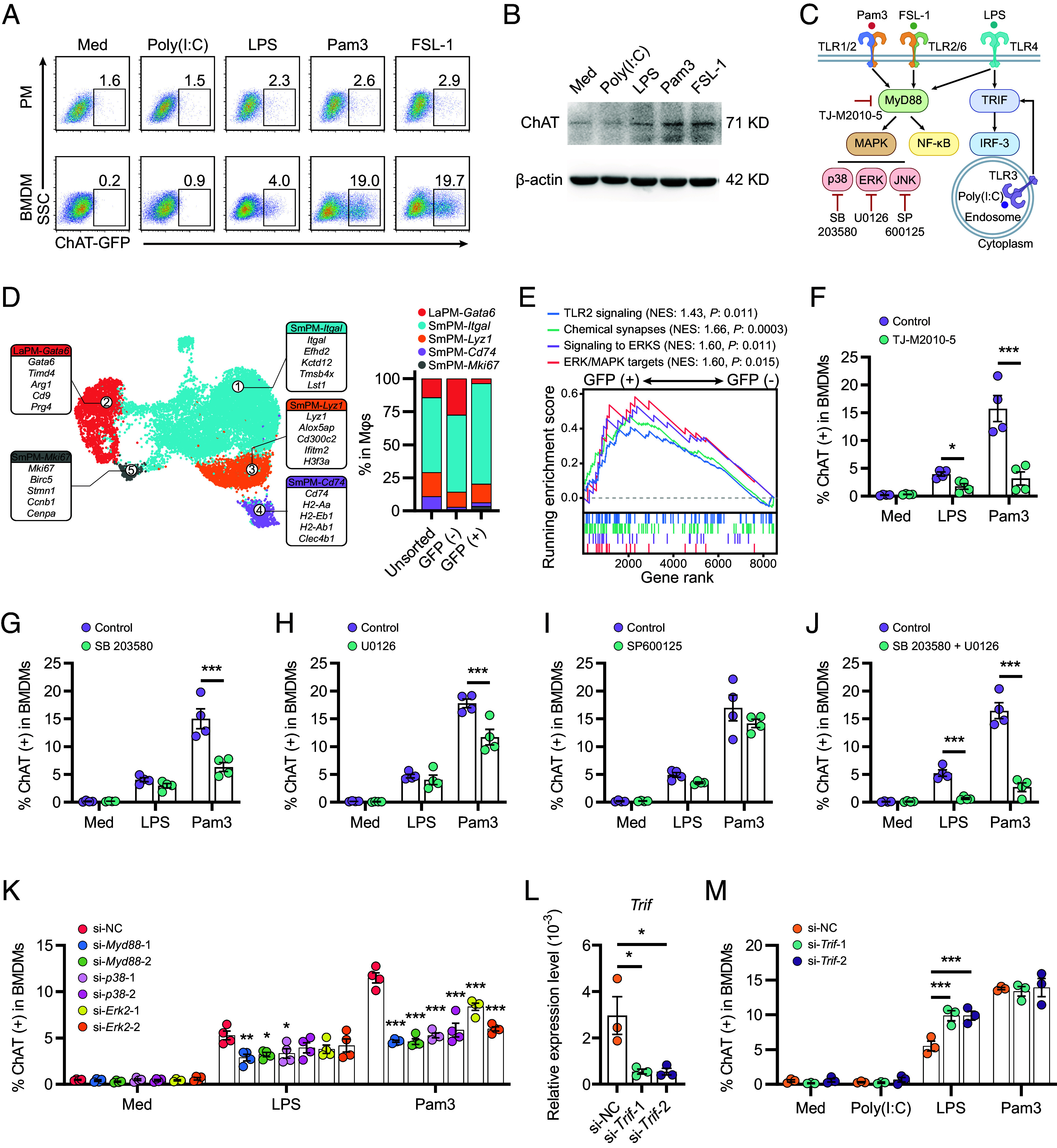
Induction of ChAT expression in BMDMs via MyD88-p38/ERK-dependent TLR signaling. (*A*) Representative flow cytometry results of GFP-expressing cells in resident peritoneal Mϕs (PMs) or BMDMs from ChAT-GFP mice stimulated with specified TLR ligands. (*B*) Immunoblotting of ChAT expression in BMDMs from wild-type B6 mice following specified treatments for 24 h. (*C*) A schematic diagram of TLR signaling and the inhibitors used in this study. (*D*) UMAP of scRNA-seq data obtained from peritoneal lavage cells (*Left*), and the frequencies of immune cell clusters (*Right*) within the unsorted, GFP^+^ and GFP^−^ Mϕs were assessed. (*E*) Gene set enrichment analysis (GSEA) of TLR2 signaling (R-MMU-168179), chemical synapses (R-MMU-112315), signaling to ERKS (R-MMU-198753), and ERK/MAPK targets (R-MMU-187687) activities. The top portion of the plot shows the running enrichment score as the analysis walks down the ranked list. The bottom portion of the plot shows where the member genes of the gene set appear in the ranked list. The normalized enrichment score (NES) and the nominal *P* values are shown. (*F–J*) BMDMs were treated with the MyD88 inhibitor TJ-M2010-5 (30 μM; *F*), the p38 inhibitor SB 203580 (25 μM; *G*), the ERK inhibitor U0126 (25 μM; *H*), the JNK inhibitor SP600125 (10 μM; *I*) or in combination with SB 203580 and U0126 (*J*) for 24 h. Subsequently, the GFP expression levels were assessed by flow cytometry. (*K*) BMDMs were treated with LPS or Pam3 for 24 h following transfection with si-*Myd88*-1, si-*Myd88*-2, si-*p38*-1, si-*p38*-2, si-*Erk2*-1, si-*Erk2*-2, or nonspecific siRNA control (si-NC). Subsequently, the GFP expression levels were assessed by flow cytometry. (*L*) BMDMs were treated with Pam3 for 24 h following transfection with si-*Trif*-1, si-*Trif*-2, or si-NC. The relative expression of *Trif* RNA was determined using qPCR. (*M*) The expression levels of GFP in BMDMs under indicated treatments were assessed by flow cytometry. Error bars indicate mean ± SEM. Statistics: (*F–K* and *M*) Two-way ANOVA corrected by Šidák’s test. (*L*) One-way ANOVA corrected by Dunnett’s test. *P < 0.05, **P < 0.01, and ***P < 0.001. Med, Medium.

The observation that SmPMs preferentially express *Chat*-GFP under Pam3 stimulation was further substantiated by in vitro studies. We found that resident peritoneal Mϕs (PMs), predominantly LaPMs (27), exhibited minimal GFP expression in response to various TLR agonists (Fig. 3 *A*, *Upper*). In contrast, SmPMs are MDMs originating from bone marrow (27). Consistent with this notion, bone marrow-derived Mϕs (BMDMs) exhibited pronounced upregulation of GFP expression upon stimulation with the TLR1/2 agonist Pam3 and the TLR2/6 agonist FSL-1 (Fig. 3 *A*, *Lower*) and, to a lesser extent, the TLR4 agonist LPS (Fig. 3*A* and *SI Appendix*, Fig. S3*A*). Consistent with the *Chat*-GFP reporter findings, ChAT protein expression was up-regulated in BMDMs derived from wild-type B6 mice in response to LPS, Pam3, or FSL-1 but not in response to poly(I:C) (Fig. 3*B*). Further investigation using a comprehensive panel of immune-related and neuroactive molecules, including various TLR agonists (e.g., TLR3, TLR7/8, and TLR9), neurotransmitters (e.g., corticosterone, norepinephrine, dopamine, and serotonin), and cytokines (e.g., IL-6, TNF-α, and IFN-γ), revealed that only the TLR7/8 agonist R848 and the TLR9 ligand CpG significantly induced ChAT expression in BMDMs (*SI Appendix*, Fig. S3*B*). These findings highlight the role of TLR pathways in regulating the expression of ChAT in Mϕs.

Upon ligand binding, TLRs typically initiate downstream signaling predominantly through the MyD88 adaptor protein (e.g., TLR4, TLR1/TLR2, TLR2/TLR6, TLR7/TLR8, TLR9) and/or via the TRIF-dependent pathway (TLR3, TLR4) (29) (Fig. 3*C*). The selective induction of ChAT in BMDMs by MyD88-activating stimuli (LPS, Pam3, FSL-1, R848, and CpG) and the negligible effect of poly(I:C), a TLR3-TRIF activator, indicated that MyD88 was the predominant factor involved in TLR-mediated ChAT expression (Fig. 3 *A* and *B* and *SI Appendix*, Fig. S3*B*). To reveal the role of TLR-mediated MyD88 signaling in ChAT expression, we first performed single-cell RNA sequencing (scRNA-seq) on total, GFP-negative, and GFP-positive peritoneal Mϕs following Pam3 stimulation (Fig. 3*D* and *SI Appendix*, Fig. S3*C* and Datasets S2 and S3). This single-cell transcriptomic analysis confirmed the selective enrichment of *Chat*-GFP in SmPMs. Interestingly, ChAT-expressing and ChAT-negative SmPMs did not exhibit distinct transcriptional landscapes in the uniform manifold approximation and projection (UMAP) analysis, and we found that ChAT-negative peritoneal Mϕs retained the potential to transition into ChAT-positive cells upon Pam3 stimulation (*SI Appendix*, Fig. S3*D*). Nevertheless, we identified 1,662 differentially expressed genes (DEGs) between these two populations (|log fold change| ≥ 0.25; false discovery rate ≤ 0.05; Fig. 3*D* and Dataset S3). ChAT-expressing SmPM showed a marked increase in the expression of genes involved in TLR signaling, chemical synapses (ChAT expression), and MyD88-dependent signaling to extracellular signal-regulated kinases/mitogen-activated protein kinase (ERK/MAPK) and their downstream targets (Fig. 3*E*). Consistently, in vitro antagonism of MyD88 (TJ-M2010-5; Fig. 3*F*), MAPK p38 (SB 203580; Fig. 3*G*), or ERK (U0126; Fig. 3*H*), but not JNK (SP600125; Fig. 3*I*), effectively inhibited ChAT expression in BMDMs, and combined inhibition of p38 and ERK had profound effects on LPS and Pam3-induced ChAT expression (Fig. 3*J*). These results were further supported by gene knockdown experiments using silencing RNAs (siRNAs), which showed that *Myd88* (*SI Appendix*, Fig. S3*E*)*, p38* (*SI Appendix*, Fig. S3*F*), and *Erk2* (*SI Appendix*, Fig. S3*G*) knockdown in BMDMs reduced the Pam3-induced ChAT expression (Fig. 3*K*).

Given the activation of MyD88 signaling by both LPS and Pam3, the relatively lower level of ChAT expression induced by LPS than by Pam3 was intriguing (Fig. 3 *A* and *B*). Considering that LPS-TLR4 stimulation activates both MyD88 and TRIF-dependent pathways (Fig. 3*C*), we hypothesized that the TRIF pathway might impede ChAT expression. As expected, *Trif* knockdown in BMDMs (Fig. 3*L*) elevated the LPS-induced ChAT expression to a level similar to that induced by Pam3 (Fig. 3*M*). These findings suggest that while TLR-MyD88-MAPK p38/ERK signaling promotes ChAT expression in MDMs, TRIF-dependent TLR signaling partially inhibits this process.

### ChAT Expression in Mϕs Promotes the Resolution of Inflammation.

**Fig. 4.**
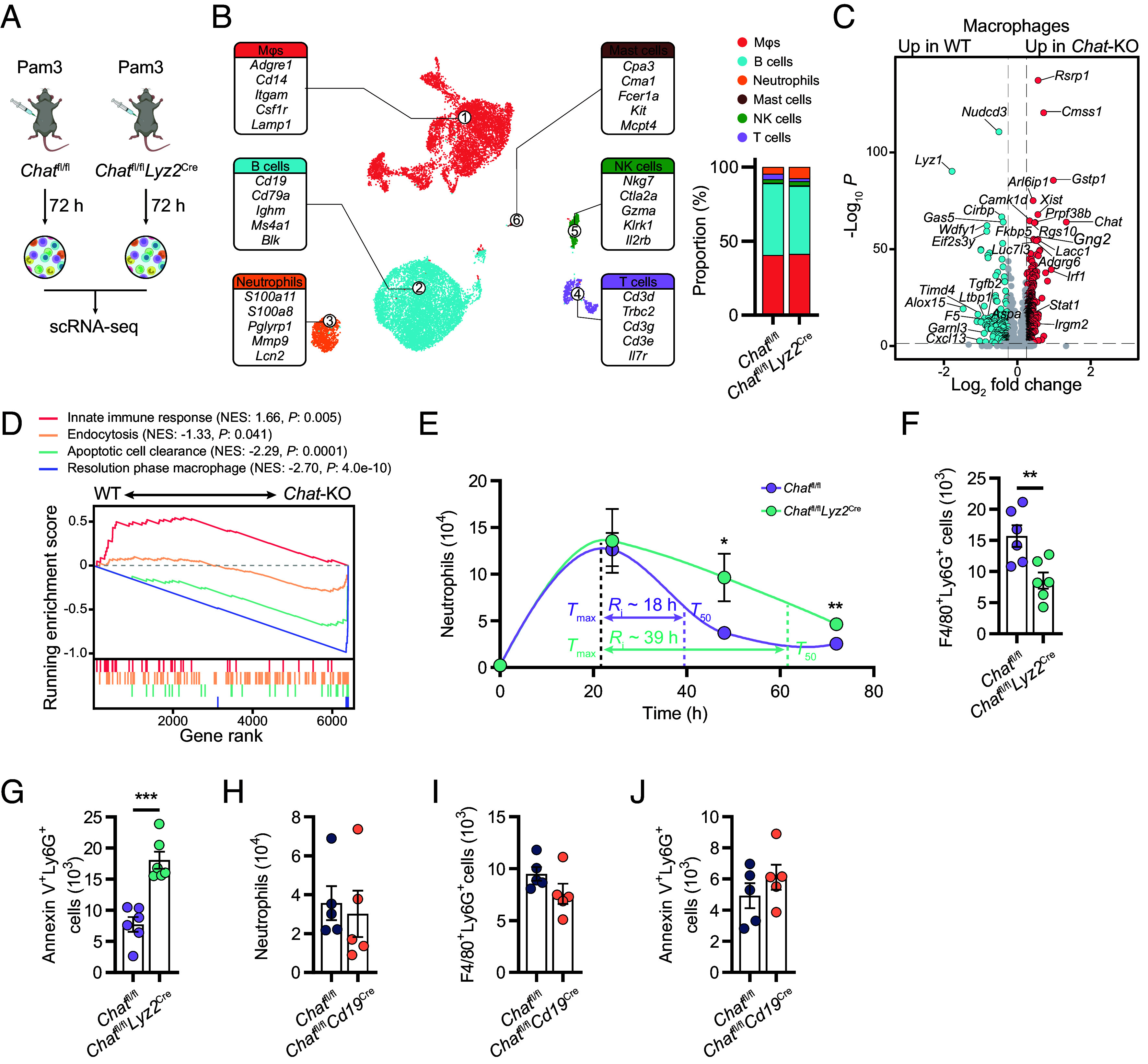
Role of ChAT-expressing peritoneal Mϕs in resolving peritoneal inflammation. (*A*) Scheme of the scRNA-seq analysis on peritoneal lavage cells from i.p. Pam3-treated *Chat*^fl/fl^ and *Chat*^fl/fl^*Lyz2*^cre^ mice. (*B*) UMAP of scRNA-seq data obtained from peritoneal lavage cells (*Left*), and the frequencies of immune cell clusters (*Right*). (*C*) Volcano plots comparing differentially expressed genes in Mϕs between WT (*Chat*^fl/fl^) and *Chat*-KO (*Chat*^fl/fl^*Lyz2*^cre^) mice. (*D*) GSEA of innate immune response (GO:0002218), endocytosis (GO:0030100), apoptotic cell clearance (GO:0043277), and resolution phase Mϕ activity (33). The top portion of the plot shows the running enrichment score as the analysis walks down the ranked list. The bottom portion of the plot shows where the member genes of the gene set appear in the ranked list. The normalized enrichment score (NES) and the nominal *P* values are shown. (*E*) Neutrophils were enumerated at different time points after i.p. Pam3 treatment in *Chat*^fl/fl^ and *Chat*^fl/fl^*Lyz2*^cre^ mice. *T*_max_ (time point when neutrophil numbers reach maximum), *T*_50_ (time point corresponding to ~50% neutrophil reduction), and *R_i_* (resolution interval, the interval between *T*_max_ and *T*_50_). (*F* and *G*) Activity of efferocytosis (F4/80^+^Ly6G^+^ cell number; *F*) and the number of remaining apoptotic neutrophils (Annexin V^+^Ly6G^+^; *G*) were assessed by flow cytometry at day 3 post-Pam3 stimulation in *Chat*^fl/fl^ and *Chat*^fl/fl^*Lyz2*^cre^ mice. (*H–J*) The neutrophil number (*H*), efferocytosis activity (*I*), and the level of remaining apoptotic neutrophils (*J*) were assessed by flow cytometry at day 3 post-Pam3 stimulation in *Chat*^fl/fl^ and *Chat*^fl/fl^*Cd19*^cre^ mice. Error bars indicate mean ± SEM. Statistics: (*E*) Two-way ANOVA corrected by Šidák’s test. (*F–J*) Student’s *t* test. *P < 0.05, **P < 0.01, and ***P < 0.001. WT, wild type; KO, knockout; i.p., intraperitoneal injection.

To elucidate the role of ChAT expression in Mϕs during the resolution of inflammation, we conducted scRNA-seq on peritoneal cell populations induced by Pam3 in *Chat*^fl/fl^*Lyz2*^cre^ cells and their control *Chat*^fl/fl^ counterparts (Fig. 4*A*). The results showed that *Chat* deletion in Mϕs was associated with increased neutrophil counts (Fig. 4*B* and Dataset S4) and a notable downregulation of genes essential for Mϕ-mediated resolution of inflammation, such as *Alox15*, *Timd4*, *F5*, *Ltbp1*, *Cxcl13*, and *Tgfb2* (30–32) (Fig. 4*C* and *SI Appendix*, Fig. S4*A* and Dataset S5). In *Chat*-deficient Mϕs, a significantly enhanced degree of active gene transcription was observed, as evidenced by greater levels of nascent (unspliced) mRNAs (*SI Appendix*, Fig. S4*B*), than that in their *Chat*-competent counterparts. Gene set enrichment analysis (GSEA) demonstrated enrichment of these mRNAs among the genes associated with innate immune responses (GO:0002218) (Fig. 4*D*), indicating a state of prolonged Mϕ activation. Concurrently, there was a profound reduction in the expression of genes associated with endocytosis (GO:0030100) and apoptotic cell clearance (GO:0043277), as well as in the expression of features typical of resolution-phase Mϕs (33), suggesting an impaired function for resolving inflammation (Fig. 4*D*).

The resolution interval (*R_i_*) is widely applied to quantify resolution speed (23, 34), which is defined as the time period between *T_max_* (the time point when neutrophil numbers reach their maximum) and *T_50_* (the time point corresponding to approximately 50% neutrophil reduction). *Chat*^fl/fl^*Lyz2*^cre^ mice exhibited a prolonged *R_i_* (*R_i_*; ~18 h and 39 h in the *Chat*^fl/fl^ and *Chat*^fl/fl^*Lyz2*^cre^ mice, respectively) and a delayed reduction in exudate neutrophils (Fig. 4*E*). The efferocytosis of apoptotic neutrophils by Mϕs was reduced by half in *Chat*^fl/fl^*Lyz2*^cre^ mice at 72 h (Fig. 4*F*), resulting in a twofold increase in the number of peritoneal apoptotic neutrophils compared to that in *Chat*^fl/fl^ mice (Fig. 4*G*). In contrast, *Chat* deficiency in B cells, the other main population of ChAT-expressing cells under Pam3 stimulation (Fig. 1*A*), had only a marginal, if any, impact on the neutrophil numbers (Fig. 4*H*), efferocytosis (Fig. 4*I*), and peritoneal apoptotic neutrophil counts (Fig. 4*J*). Collectively, these findings underscore the critical role of ChAT expression in peritoneal Mϕs, rather than B cells, in facilitating inflammation resolution.

### ACh Enhances Mϕ Phagocytosis through Nicotinic ACh Receptors.

**Fig. 5.**
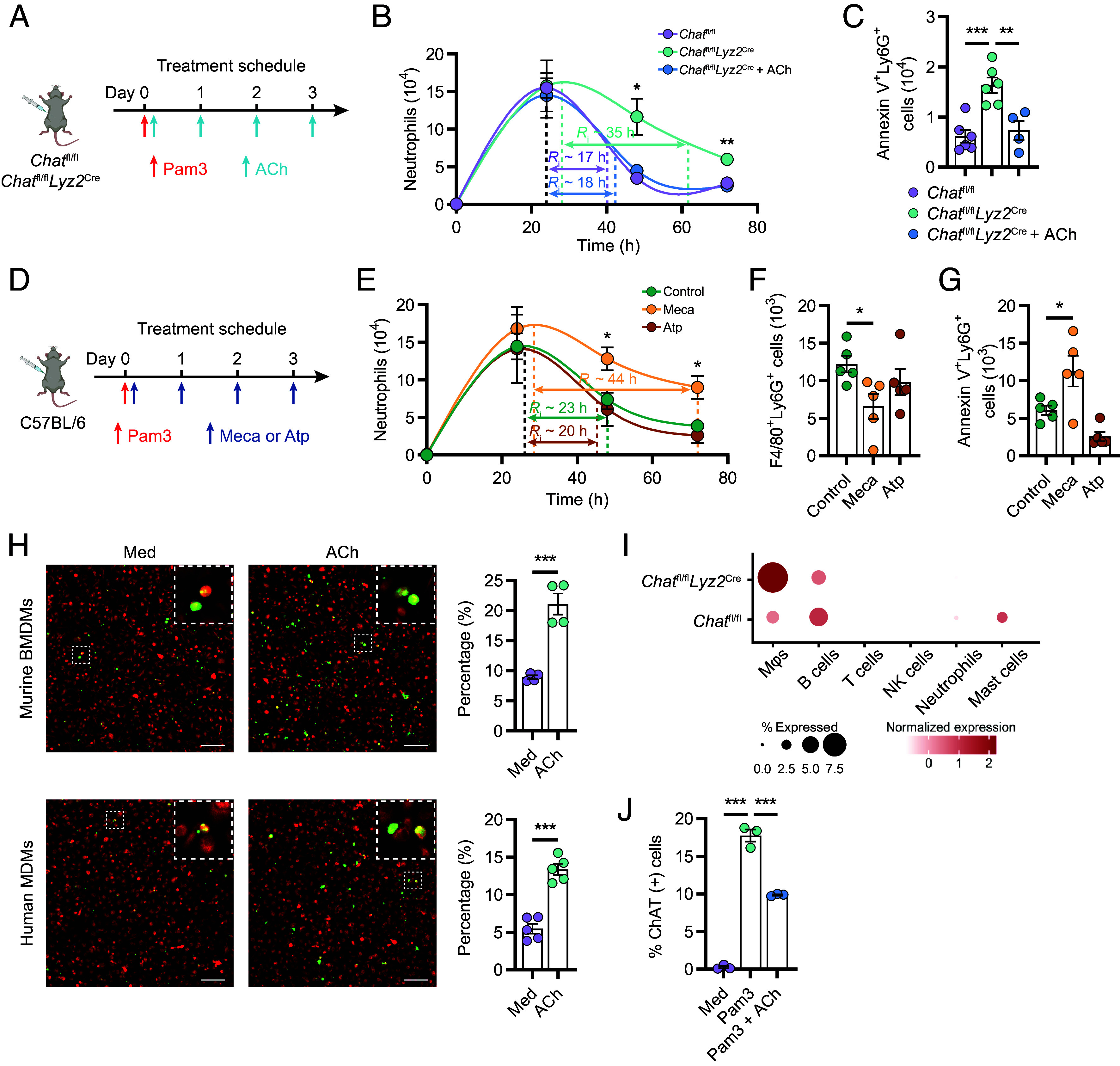
ACh enhances Mϕ phagocytosis through nicotinic receptors. (*A*) Pam3 was injected i.p. to *Chat*^fl/fl^, *Chat*^fl/fl^*Lyz2*^cre^ mice, with or without daily supplementation of ACh at a dose of 5 mg/kg. (*B*). Neutrophils were enumerated at different time points following specified treatments. (*C*) The number of apoptotic neutrophils was assessed by flow cytometry at day 3 post-Pam3 stimulation in *Chat*^fl/fl^ and *Chat*^fl/fl^*Lyz2*^cre^ mice. (*D*) Wild-type B6 mice were injected i.p. with Pam3, followed by daily administration of muscarinic receptor inhibitor atropine (Atp; 1 mg/kg) or nicotinic receptor inhibitor mecamylamine (Meca; 1 mg/kg). (*E*) Neutrophils were enumerated at different time points following specified treatments. (*F* and *G*) Activity of efferocytosis (*F*), and the number of remaining apoptotic neutrophils (*G*) were determined by flow cytometry. (*H*) The impact of ACh (1 μM) on the uptake of latex beads-rabbit IgG-FITC complex (green) in murine BMDMs (red; *Upper*) or human MDMs (red; *Lower*) was assessed. The data are presented as the percentage of latex beads-rabbit IgG-FITC-positive cells relative to the total cell count. (Scale bar, 100 μm.) (*I*) Dot plots of scaled *Chat* expression (scRNA-Seq) in indicated cell populations from *Chat*^fl/fl^ and *Chat*^fl/fl^*Lyz2*^cre^ peritoneal lavage. (*J*) BMDMs from ChAT-GFP mice were treated with Pam3, or in combination with ACh (10 μM), for 24 h and subsequently evaluated for GFP expression. Error bars indicate mean ± SEM. Statistics: (*B* and *E*) Two-way ANOVA corrected by Šidák’s test. (*C*, *F*, *G*, and *J*) One-way ANOVA corrected by Tukey’s test. (*H*) Student’s *t* test. *P < 0.05, **P < 0.01, and ***P < 0.001. Med, Medium; i.p., intraperitoneal injection.

To investigate the role of Mϕ-derived ACh in the resolution of inflammation, we administered ACh intraperitoneally to *Chat*^fl/fl^*Lyz2*^cre^ mice following Pam3 treatment (Fig. 5*A*). Remarkably, ACh supplementation significantly reduced the *R_i_* from 35 h to 18 h, effectively normalizing both the decline of neutrophils (Fig. 5*B*) and the apoptotic neutrophils count in *Chat*^fl/fl^*Lyz2*^cre^ mice to those in ChAT-competent mice (Fig. 5*C*). These findings prompted an investigation into the specific receptors mediating the effect of ACh on Mϕ phagocytosis. MDMs were found to express a range of ACh receptors (AChRs), specifically including the M3 and M4 subunits of muscarinic AChRs and the α2, α5, α9, β1, and β2 subunits of nicotinic AChRs (*SI Appendix*, Fig. S5 *A* and *B* and Dataset S6). The intraperitoneal administration of the nicotinic receptor inhibitor mecamylamine (Meca; Fig. 5*D*), but not the muscarinic receptor antagonist atropine (Atp), led to an increase in the *R_i_* from 23 h to 44 h and a delayed reduction in exudate neutrophils (Fig. 5*E*), diminished efferocytosis (Fig. 5*F*), and elevated apoptotic neutrophil counts (Fig. 5*G*). Additionally, ACh significantly enhanced the phagocytosis of latex bead-rabbit IgG-FITC complexes by both murine BMDMs and human blood MDMs, indicating that ACh generally enhanced Mϕ phagocytic activity (Fig. 5*H*).

Interestingly, despite the removal of the fourth and fifth exons from the *Chat* transcripts and the absence of ChAT protein expression in the Mϕs from the *Chat*^fl/fl^*Lyz2*^cre^ mice, an unexpected increase in the number of mRNA reads corresponding to the remaining exons was observed in both the scRNA-seq and bulk RNA-seq data (Fig. 5*I* and *SI Appendix*, Fig. S5 *C* and *D* and Dataset S6). Moreover, the addition of ACh to murine BMDM cultures decreased *Chat*-GFP expression in these cells (Fig. 5*J*). Therefore, these data suggest that environmental ACh negatively affects ChAT expression in Mϕs, serving as a negative feedback mechanism to fine-tune Mϕ functions in regulating inflammation process.

## Discussion

Recent advances have underscored the critical roles of the nervous system and neurotransmitters in modulating immune responses under both physiological and pathological conditions (35–41). In the present study, our findings reveal a unique role of Mϕs as cholinergic immune cells that emerge in the resolution phase of acute inflammation. Derived from peripheral monocytes, these Mϕs up-regulate ChAT expression via the TLR-MyD88-MAPK p38/ERK signaling pathway. Importantly, the absence of *Chat* in Mϕs, rather than in B lymphocytes, led to delayed resolution of inflammation. This finding highlights the critical role of Mϕ-derived ACh in orchestrating the resolution of inflammation.

ChAT expression and ACh synthesis in immune cells have been well documented in lymphocytes, including T cells and B cells, and to a lesser extent, in NK cells (15–17). Recently, ChAT expression has also been discovered in the myeloid compartment of the immune system. A subset (approximately 4%) of adipose-resident Mϕs could up-regulate ChAT expression for adaptive thermogenesis upon acute cold exposure (18). However, the role of ACh-synthesizing myeloid cells in immune responses and their impact on inflammation and immunity remain largely unexplored. Our study addresses this gap by revealing a notable proportion of peritoneal Mϕs expressing ChAT in the resolution phase of TLR ligands-induced peritonitis and bacterial infections. Unlike adipose-resident Mϕs, peritoneal resident LaPMs exhibit limited ChAT upregulation in response to these stimuli. In contrast, approximately 40% of the SmPMs displayed robust ChAT expression in response to Pam3 stimulation, and similar percentages were observed in conditions induced by TLR ligands and bacteria that activate MyD88 signaling. This inducible ChAT expression kinetics in SmPMs contrasts with the relatively stable ChAT expression in peritoneal B cells. These SmPMs, which have a distinct transcriptional profile independent of *Gata6* (42, 43) and lower expression levels of typical LaPM markers such as F4/80, CD11b, and Tim-4, are proposed to constitute a group of heterogeneous, recruited MDMs. Consistent with this notion, BMDMs but not isolated peritoneal LaPMs up-regulated ChAT expression in response to multiple TLR signaling pathways. These findings demonstrate the intrinsic capacity of specific Mϕ subsets to synthesize ACh, thus expanding our understanding of the non-neuronal cholinergic system within the immune landscape.

The role of ACh in the immune system is multifaceted and appears paradoxical. While immune cell-derived ACh has been shown to protect mice against sepsis and reduce the severity of experimental autoimmune encephalomyelitis, this molecule also enhances inflammation against viral infections and boosts immune responses to intestinal pathogens (15, 17, 21, 44). This complexity reflects the diverse roles of ACh in immune modulation and the variety of neurotransmitter receptors expressed by immune cells. Our study found that deleting *Chat* in Mϕs led to a delay in inflammation resolution, which was reversed by ACh supplementation. This underscores the importance of Mϕ-derived ACh in the inflammation resolution process. Interestingly, the conditional *Chat* knockout prominently altered the transcriptional landscape of Mϕs, suggesting an autocrine and/or paracrine mechanism of action. One key finding is that the absence of *Chat* in Mϕs impaired the efferocytosis of apoptotic neutrophils, thereby slowing the reduction in the number of exudate neutrophils. Moreover, the administration of nicotinic receptor inhibitors further suggested the importance of ACh-nicotinic receptor interaction in efferocytosis, as it led to reduced clearance of apoptotic neutrophils by Mϕs. Intriguingly, despite B cells being a notable ChAT-expressing population within the peritoneal cavity, *Chat* deficiency in B cells did not significantly alter the resolution process. These findings highlight the cell type-specific roles of ACh in immune regulation. Taken together, these insights into the role of Mϕ-derived ACh in immune regulation not only unravel the complexities of cholinergic signaling but may also open broad avenues for therapeutic interventions.

In immune cells, the regulation of ChAT expression is complex and diverse, often linked to cellular activation. ChAT expression in T cells is modulated by TCR engagement and cytokines such as IL-21, and in B cells, it is triggered by antigen receptor activation or MyD88-dependent signaling (13, 16, 21). Our study extends this understanding to peritoneal Mϕs and demonstrates that ChAT expression in peritoneal Mϕs is regulated via a MyD88-dependent pathway involving MAPK p38 and ERK signaling. Interestingly, although LPS effectively activates the TLR-MyD88 pathway, it only marginally induces ChAT expression in Mϕs. This difference could be attributed to the engagement of the TRIF-dependent TLR signaling pathway by LPS, which may partially counteract ChAT expression. Moreover, our tests of a range of neurotransmitters and cytokines did not reveal significant ChAT expression in BMDMs, suggesting a highly selective induction mechanism in Mϕs.

Our study identified distinct ChAT expression patterns in SmPMs and LaPMs. SmPMs but not LaPMs are the predominant peritoneal Mϕ population that express ChAT in response to TLR agonists and bacterial infection. This divergence suggests differing functional roles and activation thresholds among Mϕ subsets, an aspect that merits further investigation. Intriguingly, we observed a paradoxical increase in *Chat* gene transcription following ChAT protein depletion coupled with a reduction in ChAT expression upon exposure to elevated extrinsic ACh. This phenomenon suggests the presence of a sophisticated negative feedback mechanism involved in ChAT regulation, highlighting the complexity and adaptability of the cholinergic system during the immune response. Future research aimed at deciphering the molecular process regulating ChAT expression across lymphoid and myeloid cell populations could offer crucial insights into the diverse and context-dependent regulation of immune-neural cross talk.

In summary, our research identified a significant population of ChAT-expressing Mϕs that are crucial for resolving inflammation. These findings enhance our understanding of the intricate interplay between cholinergic signaling and Mϕ-mediated inflammatory responses, enriching the broader landscape of the involvement of the non-neuronal cholinergic system. A better understanding of these cholinergic Mϕs may illuminate different pathways for therapeutic exploration, potentially revolutionizing treatment strategies for various immune-related conditions.

## Materials and Methods

### Mice.

All mice were maintained under specific pathogen-free conditions in the animal facilities of Sun Yat-sen University (Guangzhou, China). Wild-type C57BL/6 mice (5 to 8 wk of age; Cat# GDMLAC-07) were purchased from Guangdong Medical Laboratory Animal Center (Guangzhou, China). C57BL/6JGpt-Lyz2^em1Cin(iCre)^/Gpt (Lyz2 -icre; Cat# T003822) mice and C57BL/6JGpt-Cd19^em1Cin(P2A-iCre)^/Gpt (Cd19-icre; Cat# T003785) mice were purchased from GemPharmatech (Nanjing, China). B6.Cg-Tg(RP23-268L19-EGFP)2Mik/J (*Chat*-GFP; Cat# 007902, RRID: IMSR_JAX:007902) mice and B6;129-*Chat^tm1Jrs^*/J (*Chat*^flox^; Cat# 016920, RRID: IMSR_JAX:016920) mice were purchased from The Jackson Laboratory. All animal experiments were done in accordance with approved protocols from the institutional animal care and use committees (IACUCs) of Sun Yat-sen University, according to national and institutional guidelines.

### Culture of BMDMs, Peritoneal Resident Mϕs (PMs), and Human MDMs.

For BMDMs culture, tibia and femur bone were harvested bilaterally and bone marrow was flushed out using a syringe filled with phosphate-buffered saline (PBS) containing 1% fetal bovine serum (FBS; Cat# 10270106; Thermo Fisher Scientific) and 2 mM EDTA. Bone marrow cells were plated in Iscove’s Modified Dulbecco’s Medium (Cat# 12200036; Thermo Fisher Scientific) supplemented with 10% FBS and 10 ng/mL M-CSF (Cat# 51112-MNAH; Sino Biological) for 7 d in order to obtain differentiated BMDMs. For stimulation, BMDMs obtained at the end of the 7-d culture were treated with indicated stimulation for an additional 24 h prior to cell harvesting.

For peritoneal resident Mϕs culture, we implemented a protocol to isolate and culture these cells. Briefly, after killing the mice, the abdominal area was disinfected with 70% ethanol. A midline incision was made, and 10 mL of PBS was injected into the peritoneal cavity. The peritoneal lavage cells were collected, pelleted by centrifugation, and resuspended in RPMI 1640 medium (Cat# C11875500BT; Thermo Fisher Scientific) supplemented with 10% FBS. After a 1 h incubation at 37 °C, the cells were washed five times with 1 mL of warm PBS to remove nonadherent cells, followed by a 24 h resting period. The cells were stimulated with indicated stimulation for 24 h prior to cell harvesting, and GFP expression was assessed by flow cytometry. The detailed activation conditions are listed in *SI Appendix*, Table S2.

For human MDMs culture, monocytes were isolated from peripheral blood and cultured as previously described (45, 46). Briefly, the buffy coats were collected from the blood of healthy individual donors to the Guangzhou Blood Center (Guangzhou, China). Primary blood mononuclear cells were isolated by Ficoll density gradient centrifugation at 450 g. CD14^+^ monocytes were then purified using magnetic beads (Cat# 130-050-201; Miltenyi Biotec). For Mϕ differentiation, 8 × 10^5^ monocytes were cultured per well in 24-well plates (Cat# P24-1.5H-N; Cellvis), using RPMI 1640 medium supplemented with 10% FBS and 100 ng/mL of human recombinant M-CSF (Cat# 11792-HNAH; Sino Biological). Seven days later, the cells were replenished with fresh media without M-CSF and subjected to phagocytic activity testing as described below.

### Bacteria.

*E. coli* (ATCC 25922) or *S. aureus* (ATCC 29213) was grown in Tryptic Soy Broth (Cat#211825; BD Bioscience) and harvested at mid-log phase and washed twice with sterile saline before inoculation into mouse peritoneal cavity.

### Peritonitis Induction.

For the sterile peritonitis model, mice received a single intraperitoneal injection of LPS from *E. coli* O111:B4 (1.25 mg/kg) or Pam3CSK4 (Pam3; 5 mg/kg). For the bacterial peritonitis model, mice were injected intraperitoneally with 100 μL of a suspension of live *E. coli* (1 × 10^6^ CFU per mouse) or *S. aureus* (1 × 10^6^ CFU per mouse).

### Flow Cytometry.

For the analysis of BMDMs, single-cell suspensions were obtained by incubating BMDMs in 5 mM EDTA at 37 °C for 5 min and then resuspended in FACS buffer (1% FBS and 2 mM EDTA in PBS) and stained with antibodies against CD45, CD11b, and F4/80. For the peritoneal immune cell analysis, monoclonal antibodies against CD45, CD11b, Ly6G, Ly6C, F4/80, CD3, and B220 were used. After washing three times, cells were resuspended in 200 µL of FACS buffer. Flow cytometry acquisition was performed on a Cytoflex S flow cytometer (Beckman Coulter). Finally, the data were analyzed using FlowJo software (TreeStar). Detailed descriptions of the antibodies used for flow cytometry are provided in *SI Appendix*, Table S3.

### RT-PCR.

The total RNA of BMDMs was extracted by TRIzol reagent (Cat# 15596018; Thermo Fisher Scientific) according to the manufacturer’s protocol. Total RNA was used to generate cDNA with All-in-One RT Master Mix (Cat# G492; Applied Biological Material). Real-time qPCR was performed using the SYBR Green qPCR Master Mix (Cat# B21203; Bimake). All reactions were conducted using a LightCycler 480 Instrument (Roche) in triplicate. For detection of AChRs by RT-PCR, cDNA was obtained as described above. Bands of expected sizes from electrophoresis gels were used to retrieve DNA for sequencing verification of the amplicon identities. The sequences of primers used are listed in *SI Appendix*, Table S4.

### Small Interfering RNA (siRNA) Transfection.

siRNAs (GenePharma) were mixed with jetPRIME transfection reagent (Cat# 101000046; Polyplus-transfection) according to the manufacturer’s instructions and added to the BMDM culture at a final concentration of 20 nM. After incubation with the siRNA complex for 12 h, BMDMs were stimulated with LPS or Pam3 for 24 h as indicated in the figure legends. Details of the siRNA sequences are listed in *SI Appendix*, Table S5.

### Immunoblotting.

BMDMs were washed once with PBS and lysed in an ice-cold lysis buffer [62.5 mM (pH 6.8) Tris-HCl, 10% (w/v) glycerol, 50 mM dithiothreitol, 2% (w/v) sodium dodecyl sulfate, and 0.01% (w/v) bromophenol blue in ultrapure water]. Denatured proteins were separated on a 10% SDS-PAGE gel. The proteins were then transferred onto polyvinylidene fluoride membrane and blotted with the indicated antibodies. Following staining with HRP-coupled secondary antibodies, the proteins were visualized using the SuperSignal West Pico Chemiluminescent Substrate (Cat# 34580; Thermo Fisher Scientific). The following antibodies were used in immunoblotting: β-actin (Cat# BM0627; Boster Biological Technology); ChAT (Cat# 27269; Cell Signaling Technology).

### UPLC–MS/MS.

GFP-negative and GFP-positive peritoneal Mϕs sorted from FACS were washed three times with PBS containing 0.1% pyridostigmine bromide. Subsequently, cell pellets were snap-frozen in liquid nitrogen and stored at −80 °C until analysis. To detect ACh in peritoneal lavage fluid from Pam3-treated (72 h) *Chat*^fl/fl^ and *Chat*^fl/fl^*Lyz2*^cre^ mice, peritoneal cavities were lavaged with 1 mL of sterile PBS containing 0.1% pyridostigmine bromide. Peritoneal lavage fluid was collected and centrifuged (380 g, 10 min) to remove cells. The remaining supernatant is centrifuged at high speed (12,000 g, 10 min). Following centrifugation, supernatants were snap-frozen in liquid nitrogen and stored at −80 °C until analysis. Samples were spiked with Tolbutamide internal standard (Cat# 64-77-7; Sigma–Aldrich) and analyzed by LC-tandem MS on an AB 6500Plus mass spectrometer (AB Sciex, United States) equipped with an AB EXIONLC AD instrument (AB Sciex, United States).

### ACh and AChR Inhibitor Treatment.

For ACh treatment, *Chat*^fl/fl^ and *Chat*^fl/fl^*Lyz2*^cre^ mice were injected intraperitoneally with Pam3, followed by intraperitoneal injection of ACh (5 mg/kg) 0.5 h later, and continued daily for the duration of the experiment. For AChR inhibitor treatment, wild-type B6 mice were injected intraperitoneally with Pam3, followed by intraperitoneal injection of muscarinic receptor inhibitor atropine (Atp; 1 mg/kg) or nicotinic receptor inhibitor mecamylamine (Meca; 1 mg/kg) 0.5 h later, and continued daily for the duration of the experiment. At designated points, peritoneal exudate was collected by lavaging with 5 mL PBS. The exudate cells were subsequently used for flow cytometric analyses.

### Efferocytosis Analysis.

To assess Mϕ phagocytosis of apoptotic neutrophils in vivo, peritoneal lavage cells were harvested, labeled with anti-mouse F4/80 antibody for 30 min, fixed and permeabilized with Fixation/Permeabilization Kit (Cat# 554714; BD Bioscience), and then stained with anti-mouse Ly6G antibody. The F4/80^+^Ly6G^+^ Mϕ population was determined by flow cytometry.

### Measurement of Neutrophil Apoptosis.

To determine neutrophil apoptosis, peritoneal lavage cells were labeled with annexin V and an anti-Ly6G antibody. The annexin V^+^Ly6G^+^ neutrophil population was determined by flow cytometry.

### Phagocytosis Assays.

For phagocytosis assays, cells were plated to 90% confluence in 24-well glass bottom plates and pretreated with cholinesterase inhibitor pyridostigmine bromide (Cat# HY-B0207A; MedChemExpress) and ACh (Cat# HY-B0282; MedChemExpress) for 24 h. Subsequently, cells were assessed for phagocytic activity using the Phagocytosis Assay Kit (Cat# 500290; Cayman Chemical). Briefly, BMDMs or human MDMs were labeled with 1 μM Cell Trace Far Red (Cat# C34564; Thermo Fisher Scientific). After incubation for 20 min at 37 °C, cells were washed and then the latex beads conjugated to rabbit IgG-FITC (1:400 dilution) were added to the culture in the absence or presence of ACh. During live imaging, the cells were incubated in a stage-top incubator (Tokai Hit) at 37 °C and 5% CO_2_. Images were then obtained continuously every 20 min for 2 h using an inverted microscope (Nikon, ECLIPSE Ti2) equipped with the spinning disk unit (Yokogawa, CSU-W1) and sCMOS camera (Hamamatsu, ORCA-Fusion BT).

### RNA-Seq.

BMDMs from *Chat*^fl/fl^ and *Chat*^fl/fl^*Lyz2*^cre^ mice were treated with or without LPS, Pam3, or FSL-1 for 24 h, and then washed twice with PBS. The total RNA of treated BMDMs was extracted by TRIzol reagent according to the manufacturer’s protocol. Poly-A mRNA was extracted from total RNA using Oligo-dT beads in a NEBNext Poly(A) RNA Magnetic Isolation Module (New England Biolabs) and a RiboZero Magnetic Gold Kit (Epicentre), and RNA-Seq libraries were prepared using KAPA Stranded RNA-Seq Library Prep Kit (Illumina) according to the manufacturer’s protocols. Libraries were paired-end sequenced on a NovaSeq sequencer (Illumina). The reads were aligned to the mm10 mouse genome using *Salmon,* and the aligned data were analyzed using *R* software. The analysis was conducted following the Bioconductor RNA-seq workflow and differential gene expression was analyzed using the R package *DESeq2*.

### Single-Cell RNA-Seq and Gene Expression Quantification.

Cells from Pam3-treated mice were isolated and purified by a MoFlo XDP flow cytometer (Beckman Coulter). Preparation of gel beads in emulsion and libraries was performed with Chromium Single Cell 3′ Library & Gel Bead Kit v3 (Cat# PN-1000092; 10× Genomics), according to the manufacturer’s protocol. Cell suspensions were then diluted to target a recovery of 12,000 cells per sample in the 10× Genomics system. The generated scRNA-seq libraries were sequenced on a NovaSeq sequencer (Illumina, USA) at a depth of 30,000 to 100,000 reads per cell and sequencing saturation levels between 45% and 55%. The resultant scRNA-seq reads were aligned to a prebuilt reference (refdata-gex-mm10-2020-A, provided by 10× Genomics) and quantified using *cellranger count* (Cell Ranger pipeline version 7.0.0, 10× Genomics). Quantification of nascent (unspliced) and mature (spliced) mRNA levels was performed using the *velocyto run10x* function from python library *velocyto.py* (version 0.17.17). The gene regulatory networks and transcription factor activities were inferred using pySCENIC workflow (version 0.12.1).

### Quality Control and Batch Correction.

Quality control was performed separately for each dataset. Cells were filtered based on the number of expressed genes (generally >500 genes detected), mitochondrial gene content (<10%), and hemoglobin gene expression (<1%). Additionally, doublets detected by simulation using the *scDblFinder* package were also filtered. After the filtering, we obtained a total of 29,496 cells, as follows: ChAT-GFP-positive Mϕs, 5,228 cells; ChAT-GFP negative Mϕs, 5,893 cells; *Chat*^fl/fl^ peritoneal cells, 8,835 cells (including 3,579 Mϕs); *Chat*^fl/fl^*Lyz2*^cre^ peritoneal cells, 9,540 cells (including 3,936 Mϕs). We applied a batch correction procedure to integrate the scRNA-seq data for analysis using *Seurat* package (v4). In brief, *SCTransform* normalization (vst.flavor = “v2”), *SelectIntegrationFeatures*, *PrepSCTIntegration*, *FindIntegrationAnchors*, and *IntegrateData* commands were sequentially used to achieve data integration.

### Dimensionality Reduction and Unsupervised Clustering Analysis.

To select features for the PCA, we used highly variable genes identified by *SelectIntegrationFeatures*. In order to focus the PCA on genes that are more likely to reflect the underlying biological differences between cell types or states, histone, hemoglobin, mitochondrial, and ribosomal genes, along with noncoding RNAs, artifacts, and pseudogenes, were excluded. Normalized expression values were subjected to PCA using with Seurat v4 function *RunPCA*. The leading 30 PCs were used to calculate the uniform manifold approximation and projection (UMAP) embedding using *RunUMAP* function. These 30 PCs were also used for cell clustering through embedding cells into a k-nearest neighbors graph structure using *FindNeighbors* function in PC space and for calculating Louvain clusters using *FindClusters*. We examined the resolution parameters with the help of the *clustree* package and altered the resolution to 0.06 in *FindClusters*.

### Single-Cell Differential Gene Expression Analysis and GSEA.

To find differentially expressed genes and to find marker genes, we used the *FindMarkers* function in Seurat (with a two-sided Wilcoxon rank-sum test or ROC analysis on the SCT normalized data). We considered genes that were expressed in >1% of cells and with Bonferroni-adjusted *P* < 0.05 as differentially expressed genes. For GSEA, we applied *fgsea* package, implemented in R, to the sorted gene fold-changes generated by *FindMarkers* function from Seurat. The gene sets used in this study were drawn from mouse collection of the Molecular Signature Database (CP:REACTOME and GO:BP subcategories), including TLR2 signaling (R-MMU-168179), chemical synapses (R-MMU-112315), signaling to ERKS (R-MMU-198753), ERK/MAPK targets (R-MMU-187687), innate immune response (GO:0002218), endocytosis (GO:0030100), and apoptotic cell clearance (GO:0043277). We provide two statistics in the GSEA plots: the normalized enrichment score (NES) and the nominal *P* value (47).

### LEGENDScreen and InfinityFlow Pipeline.

Mouse peritoneal lavage cells from control, LPS-, or Pam3-treated mice were obtained by washing the peritoneal cavity with ice-cold PBS four times. Erythrocytes were removed by incubating with red blood cell lysis buffer (Cat# NH4CL2009; TBD Science) at 1 min on ice. Each sample was separately stained with different anti-CD45 conjugates (as indicated in *SI Appendix*, Table S6). Following staining, cells were washed twice with PBS and then combined into a single pool of cells. Cells were then resuspended at 20 × 10^6^ cells/mL in PBS + Zombie NIR Live/Dead stain (Cat# 423106; BioLegend) at 2 μL/mL and incubated for 20 min at room temperature. Following staining, a 10-fold volume of Cell Staining Buffer (BioLegend) was added to neutralize any unbound dye, and cells were centrifuged at 300 g for 5 min. Cells were resuspended at 20 × 10^6^ cells/mL in Cell Staining Buffer, and a master mix of the indicated Backbone antibody panel (*SI Appendix*, Table S6) at 20 × 10^6^ cells/mL, and incubated for 30 min at 4 °C. Following Backbone staining, cells were washed twice and resuspended at 20 × 10^6^ cells/mL in Cell Staining Buffer, and 75 μL was added to each well of the LEGENDScreen plates (Cat# 700009; BioLegend). After staining for 30 min, plates were washed and fixed. Flow cytometry acquisition was performed on a Cytoflex S flow cytometer using CytExpert software, and data were subsequently processed through the *InfinityFlow* pipeline implemented as an R package (version 1.10.0). We applied the *infinity_flow* function of this package using a prediction_events_downsampling parameter of 2,500 and other default parameters. The output concatenated FCS file was further analyzed using FlowJo software to debarcode the pooled data into six sample groups: control GFP-negative, control GFP-positive, LPS GFP-negative, LPS GFP-positive, Pam3 GFP-negative, and Pam3 GFP-positive. The separated fcs files were then downsampled to ~10,000 cells per sample and subject to further analysis in *R*. Data preprocessing, *FlowSOM* clustering, and *ConsensusClusterPlus* metaclustering were performed using the *CATALYST* package (version 1.24.0) and T-distributed stochastic neighbor embedding (TSNE) was calculated using the *scater* package (version 1.28.0), followed by subclustering using a two-step procedure with the *clusterCells*(BLUSPARAM=TwoStepParam) function implemented in *scran* package (version 1.28.2). For differential expression analysis, we applied the *scoreMaerkers* function from the *scran* package.

## Supplementary Material

Appendix 01 (PDF)

Dataset S01 (XLSX)

Dataset S02 (XLSX)

Dataset S03 (XLSX)

Dataset S04 (XLSX)

Dataset S05 (XLSX)

Dataset S06 (XLSX)

## Data Availability

All original data supporting the findings of this study, including scRNA-seq and bulk RNA-seq, are available from the China National Genebank Database (CNGBdb) under accession number CNP0005278 (48). Codes for data analyzing and generating specific figures have been deposited to GitHub and can be accessed at https://github.com/ChongEdwardWu/PNAS_2024_ChEM (49). All other data are included in the manuscript and/or supporting information.
