# Supplementary material for: Cholinergic macrophages promote the resolution of peritoneal inflammation
Source: Proc Natl Acad Sci U S A. 2024 Jun 26;121(27):e2402143121. doi: 10.1073/pnas.2402143121 (PMC11228479; doi:10.1073/pnas.2402143121)
Supplement: Supplementary file 1 — Appendix 01 (PDF) [file pnas.2402143121.sapp.pdf]

PNAS

Supporting Information for

Cholinergic Macrophages Promote Resolution of Peritoneal Inflammation

Shufeng Luo[1,4], Huiling Lin[2,4], Chong Wu[2,4]*, Lan Zhu[2], Qiaomin Hua[1,2], Yulan Weng[2], Lu Wang[2], Xiaoli Fan[2], Kai-Bo Zhao[2], Gaoteng Liu[2], Yuting Wang[2], Hai-Tian Chen[3], Li Xu[1], and Limin Zheng[1,2]*

[1]State Key Laboratory of Oncology in South China, Guangdong Provincial Clinical Research Center for Cancer, Sun Yat-sen University Cancer Center, Guangzhou 510060, P. R. China

[2]Guangdong Provincial Key Laboratory of Pharmaceutical Functional Genes, MOE Key Laboratory of Gene Function and Regulation, School of Life Sciences, Sun Yat-sen University, Guangzhou 510275, P. R. China

[3]First Affiliated Hospital, Sun Yat-sen University, Guangzhou 510080, P. R. China

[4]These authors contributed equally to this work.

*Corresponding authors: Chong Wu (E-mail: wuchong5@mail.sysu.edu.cn; Phone: +86-020-8411 5531) and Limin Zheng (Lead contact; E-mail: zhenglm@mail.sysu.edu.cn; Phone: +86-020-8411 2163).

This PDF file includes:

Figures S1 to S5
Tables S1 to S6
Legends for Datasets S1 to S6

Other supporting materials for this manuscript include the following:

Datasets S1 to S6

Figure S1

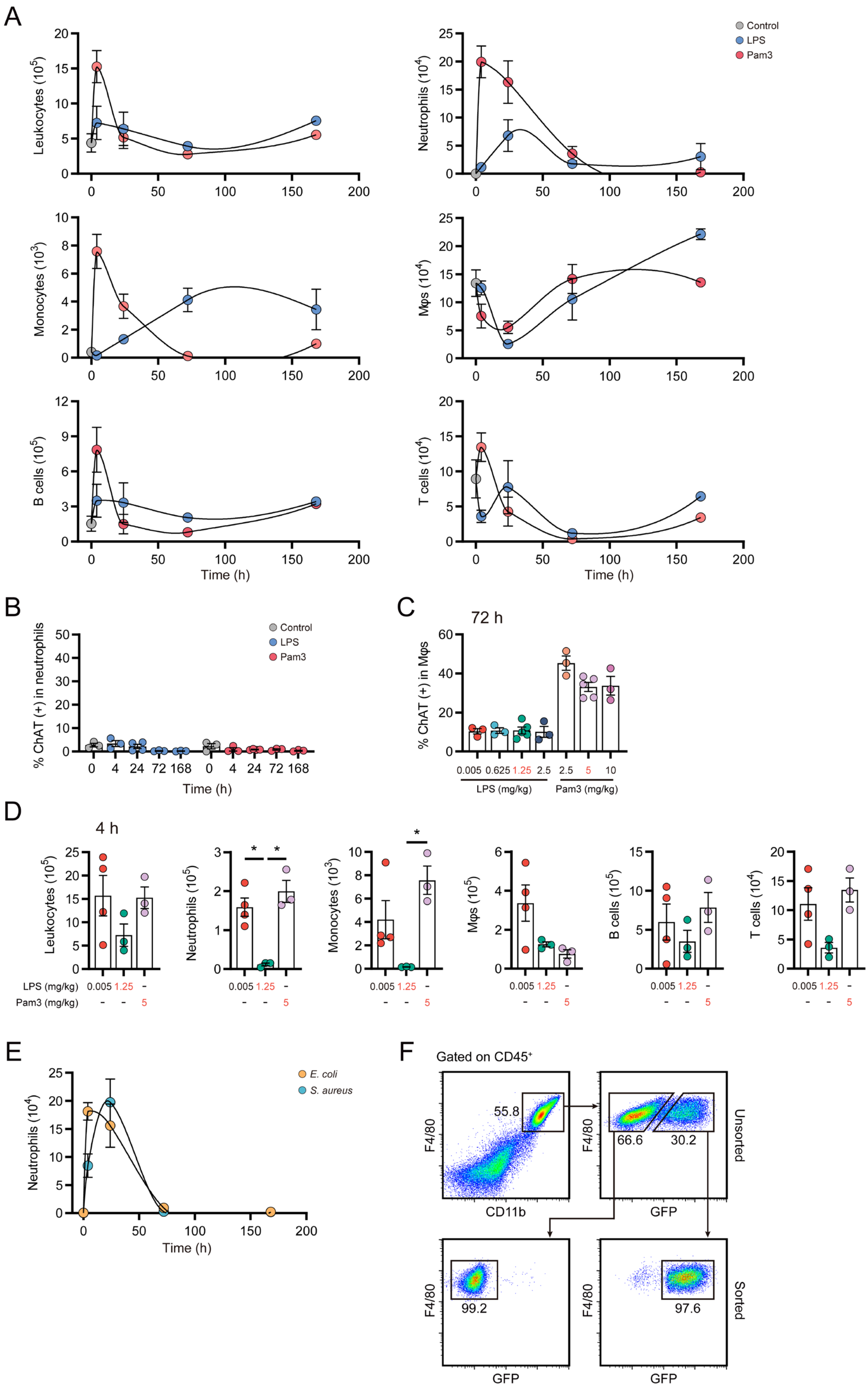
A
Control
LPS
Pam3
Leukocytes (10^5)
Neutrophils (10^4)
Monocytes (10^3)
Mφs (10^4)
B cells (10^5)
T cells (10^4)
Time (h)
B
% ChAT (+) in neutrophils
Time (h)
C
72 h
% ChAT (+) in Mφs
LPS (mg/kg)
Pam3 (mg/kg)
D
4 h
Leukocytes (10^5)
Neutrophils (10^5)
Monocytes (10^3)
Mφs (10^5)
B cells (10^5)
T cells (10^4)
LPS (mg/kg) 0.005 1.25 -
Pam3 (mg/kg) - - 5
E
E. coli
S. aureus
Neutrophils (10^4)
Time (h)
F
Gated on CD45⁺
F4/80
CD11b
GFP
55.8
66.6
30.2
99.2
97.6
Unsorted
Sorted

Figure S1. Dynamics of immune cell populations during LPS or Pam3-induced peritonitis, related to Figure 1. (**A**) LPS or Pam3 was applied to induce peritonitis in male B6 mice. Lavages were collected at indicated intervals. Leukocytes, neutrophils, monocytes, Mφs, B cells, and T cells were enumerated. (**B**) Percentages of ChAT-expressing cells in neutrophils were measured at the indicated time points following specified treatments. (**C**) Percentages of ChAT-expressing cells in Mφs were assessed by flow cytometry at day 3 after injection of LPS (0.005, 0.625, 1.25 or 2.5 mg/kg) or Pam3 (2.5, 5 or 10 mg/kg) in ChAT-GFP mice. The concentrations of LPS and Pam3 used in other experiments of this study are highlighted in red. (**D**) Leukocytes, neutrophils, monocytes, Mφs, B cells, and T cells were assessed by flow cytometry at 4 h after LPS (0.005, 1.25 mg/kg) or Pam3 (5 mg/kg) stimulation in ChAT-GFP mice. (**E**) Mice were inoculated with 10^6 CFU of either *E. coli* or *S. aureus* by intraperitoneal injection, and neutrophils were enumerated at different time points. (**F**) Flow cytometric sorting purity of the GFP^- and GFP^+ Mφs. Error bars indicate mean ± SEM. Statistics: (**D**) One-way ANOVA corrected by Tukey's test. *$P < 0.05$.

Figure S2

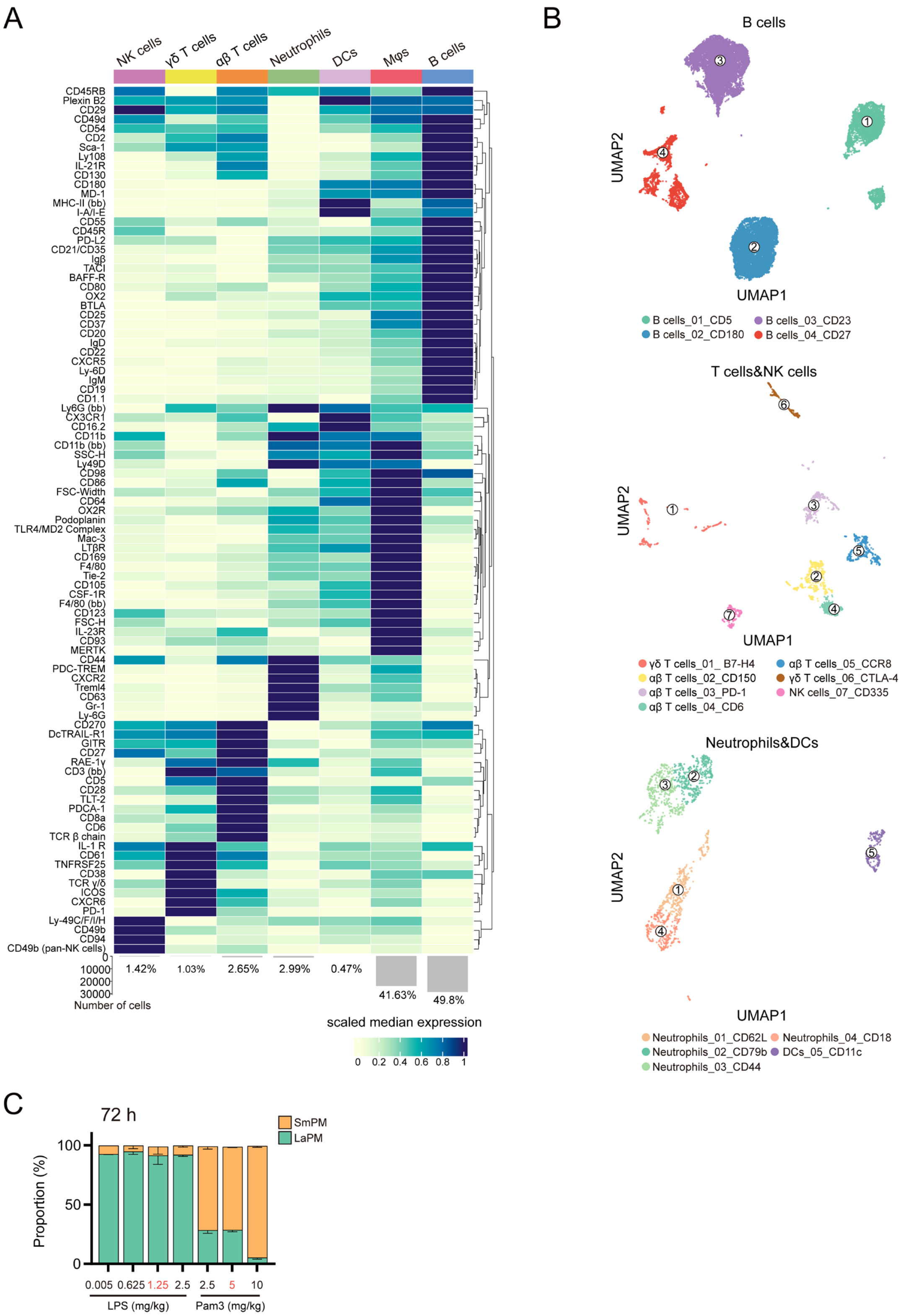

Figure S2. Phenotypic analysis of peritoneal immune cells using InfinityFlow, related to Figure 2. (**A**) Heatmap of markers differentially expressed among various subtypes of peritoneal lavage cells. (**B**) Sub-clustering of peritoneal B cells, T cells, NK cells, neutrophils, and DCs based on UMAP, highlighting distinct phenotypic cluster profiles detailed in **Dataset S1**. (**C**) The frequencies of

F4/80^{hi}Tim-4^{+} LaPMs and F4/80^{lo}Tim-4^{-} SmPMs in total Mφs were assessed by flow cytometry on day 3 after injection of LPS (0.005, 0.625, 1.25 or 2.5 mg/kg) or Pam3 (2.5, 5 or 10 mg/kg) in ChAT-GFP mice. The concentrations of LPS and Pam3 used in other experiments of this study are highlighted in red. Error bars indicate mean ± SEM.

Figure S3

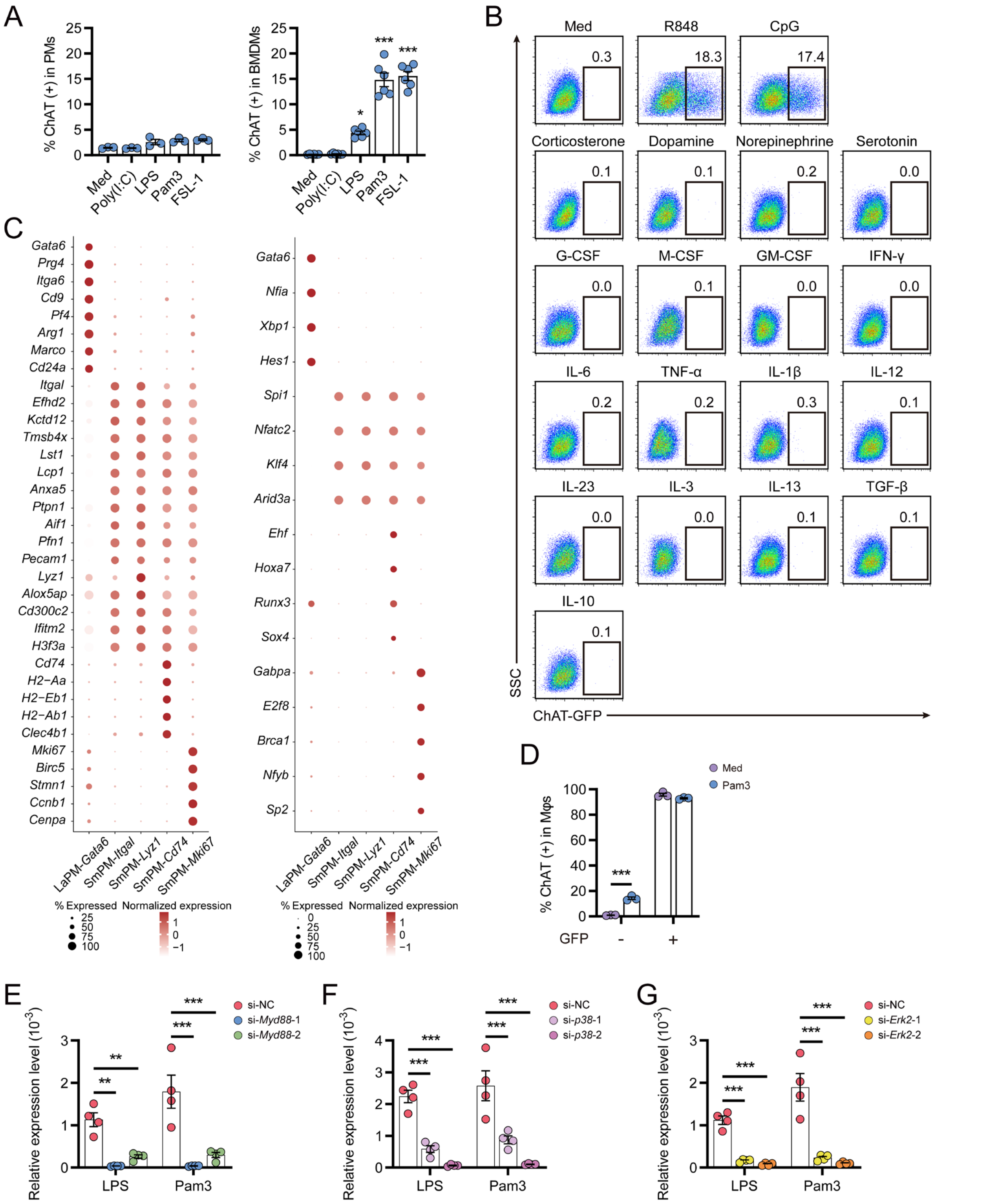

Figure S3. Regulation of ChAT expression in BMDMs via TLR signaling pathways, related to Figure 3. (**A**) Percentages of GFP-expressing cells in peritoneal resident Mφs (PMs) and BMDMs from ChAT-GFP mice following specified treatments, as determined by flow cytometry. (**B**) BMDMs from ChAT-GFP mice stimulated for 24 h with various TLR agonists, neurotransmitters, and cytokines, assessed for GFP expression by flow cytometry. (**C**) scRNA-seq analysis depicting feature genes (left panel) and transcription factors (right panel) across different peritoneal lavage cell clusters. (**D**) Percentages of GFP-expressing cells within GFP$^-$ and GFP$^+$ Mφs isolated from Pam3-treated ChAT-

GFP mice following *in vitro* stimulation with Pam3 for 24 h. (**E-G**) BMDMs were treated with LPS or Pam3 for 24 h following transfection with si-*Myd88*-1, si-*Myd88*-2, si-*p38*-1, si-*p38*-2, si-*Erk2*-1, si-*Erk2*-2, or si-NC. The relative expression of *Myd88* (**E**), *p38* (**F**) or *Erk2* RNA (**G**) was determined using quantitative PCR. Error bars indicate mean ± SEM. Statistics: (**A**) One-way ANOVA corrected by Dunnett's test. (**D-G**) Two-way ANOVA corrected by Šidák's test. *$P < 0.05$, **$P < 0.01$, ***$P < 0.001$. Med, Medium.

Figure S4

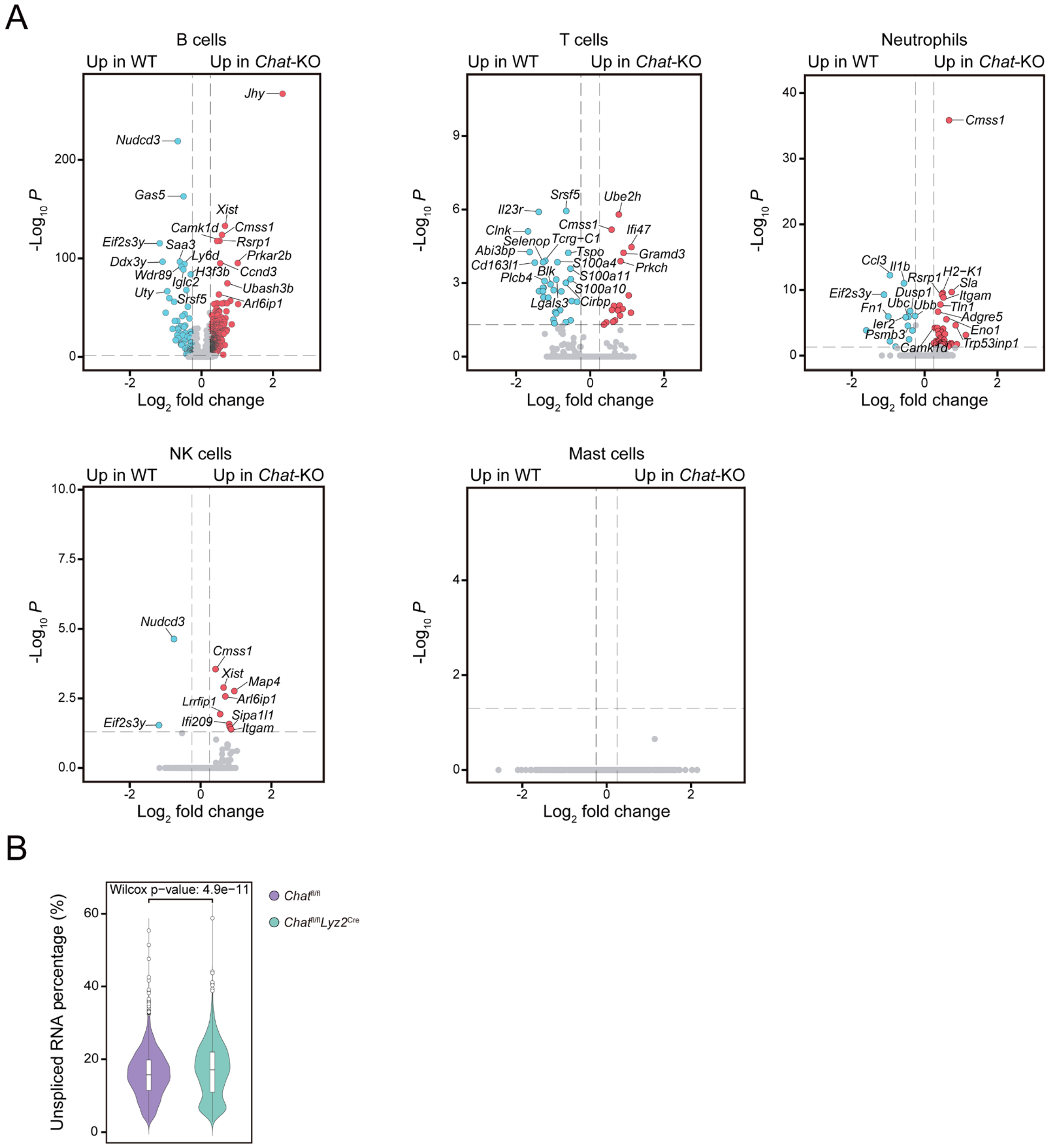

Figure S4. Differential gene expression in immune cell subsets from *Chat*$^{fl/fl}$ and *Chat*$^{fl/fl}$*Lyz2*^{cre} mice, related to Figure 4. (**A**) Volcano plots depicting differentially expressed genes in B cells, T cells, neutrophils, NK cells and mast cells from WT (*Chat*$^{fl/fl}$) compared to *Chat*-KO (*Chat*$^{fl/fl}$*Lyz2*^{cre}) mice. Key genes for each cell type are highlighted. (**B**) Proportion of unspliced RNA (scRNA-Seq) in Mφs from *Chat*$^{fl/fl}$ and *Chat*$^{fl/fl}$*Lyz2*^{cre} mice. Statistics: Wilcoxon test. WT, wild type; KO, knockout.

Figure S5

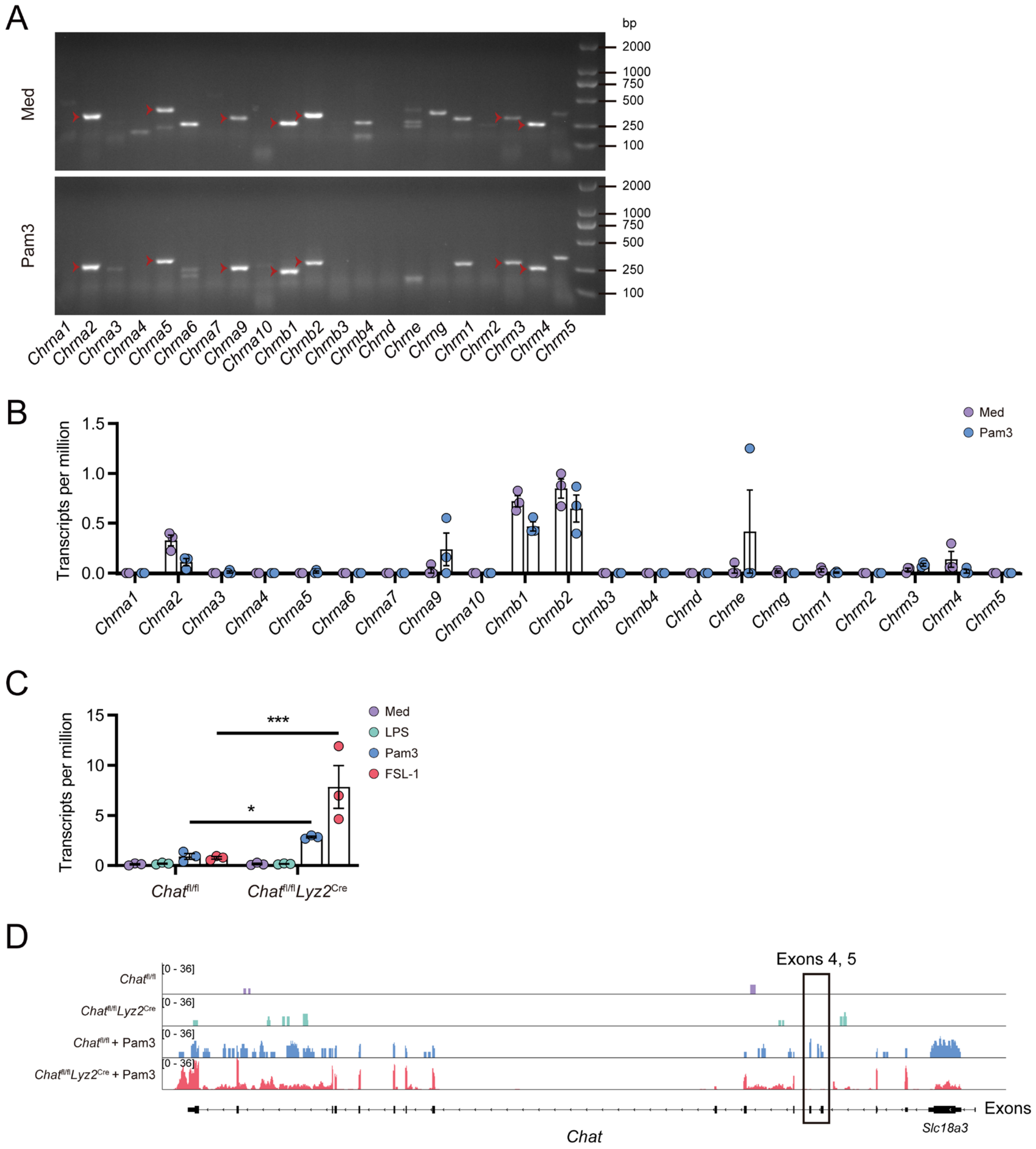

Figure S5. Analysis of ACh receptor expression and *Chat* gene regulation in Mφs, related to Figure 5. (**A and B**) Expression patterns of nicotinic and muscarinic acetylcholine receptor subunits in BMDMs under various treatments as analyzed by RT-PCR (**A**) and bulk RNA-seq (**B**). Red arrows indicate validated specific PCR products. (**C**) Transcript levels of *Chat* in BMDM following different treatments, quantified by bulk RNA-seq and presented as transcripts per million (TPM). (**D**) BMDMs from *Chat*$^{fl/fl}$ and *Chat*$^{fl/fl}$*Lyz2*^{cre} mice were cultured in either medium alone (Med) or with Pam3 for 24 h. Representative bulk RNA-seq peaks were shown as track signals in an integrative genomic viewer. Error bars indicate mean ± SEM. Statistics: (**C**) Two-way ANOVA corrected by Šidák's test. *$P < 0.05$, ***$P < 0.001$.

Table S1. The surface markers for peritoneal lavage cell subsets identification by InfinityFlow

**Marker (B cells)**	AUC	**Marker (Mφs)**	AUC	**Marker (Neutrophils)**	AUC
CD19	0.999151	F4/80-APC	0.991166	Ly-6G/Ly-6C (Gr-1)	0.998098
Ly-6D	0.998799	CD11b-PE-CF594	0.975073	Ly-6G	0.997433
CD22	0.99859	F4/80	0.946362	PDC-TREM	0.967425
CD20	0.997406	MERTK (Mer)	0.940361	Trem-like 4 (Treml4)	0.949058
IgM	0.996353	CD105	0.924017	CD63	0.947796
CD268 (BAFF-R)	0.9939	Podoplanin	0.918786	CD182 (CXCR2)	0.941937
IgD	0.993328	TLR4 (CD284)/MD2 Complex	0.916643	Ly49D	0.893341
Ly108	0.989723	lymphotoxin beta receptor (LTβR)	0.910311	Ly6G-BV785	0.877172
CD180 (RP105)	0.985332	FSC-H	0.910157	CD11b	0.865099
MD-1	0.984167	CD98 (4F2)	0.905966	CD44	0.830696
CD272 (BTLA)	0.983707	CD107b (Mac-3)	0.903071	**Marker (αβ T cells)**	AUC
CD37	0.977145	SSC-H	0.901379	CD6	0.985521
CD25	0.974652	CD64 (FcγRI)	0.899309	TCR ß chain	0.973986
CD79b (Igβ)	0.973303	CD115 (CSF-1R)	0.894414	CD5	0.955811
CD2	0.966524	CD200 (OX2)	0.889693	CD357 (GITR)	0.946586
Ly-6A/E (Sca-1)	0.959883	CD202b (Tie-2, CD202)	0.875418	CD28	0.942404
CD1d (CD1.1, Ly-38)	0.954318	CD169 (Siglec-1)	0.863168	CD27	0.937886
CD267 (TACI)	0.950072	CD86	0.83324	CD317 (BST2, PDCA-1)	0.933763
CD185 (CXCR5)	0.94488	CD123	0.826936	CD270 (HVEM)	0.913716
CD55 (DAF)	0.937045	IL-23R	0.804619	RAE-1γ	0.891151
CD45R (B220)	0.935004	FSC-Width	0.789256	DcTRAIL-R1	0.852122
CD80	0.918577	CD93 (AA4.1, early B lineage)	0.775961	TLT-2	0.819064
CD200 R (OX2R)	0.91819	**Marker (DCs)**	AUC	CD8a	0.79169
CD273 (B7-DC, PD-L2)	0.910509	I-A/I-E	0.96374	**Marker (γδ T cells)**	AUC
IL-21R	0.901801	MHC-II-BV421	0.963665	CD279 (PD-1)	0.984748
CD45RB	0.893241	Plexin B2	0.948553	CD278 (ICOS)	0.975883
CD130 (gp130)	0.867985	CX3CR1	0.903613	DR3 (TNFRSF25)	0.952171
CD21/CD35 (CR2/CR1)	0.864764	CD16.2 (FcγRIV)	0.880899	CD3-PC7	0.930274
CD49d	0.860296	**Marker (NK cells)**	AUC	CD186 (CXCR6)	0.927075
CD54	0.839304	CD49b (pan-NK cells)	0.962722	CD61	0.876878
		CD49b	0.950036	CD38	0.870059
		CD94	0.829385	CD121a (IL-1 R, Type I/p80)	0.864085
		CD29	0.801786	TCR γ/δ	0.793534
		Ly-49C/F/I/H	0.789295		

Table S2. Reagents used to induce the ChAT expression *in vitro*

Reagents	Concentration	Identifier	Supplier
LPS	100 ng/ml	Cat# L2630-25MG	Sigma-Aldrich
Pam3CSK4	1 μg/ml	Cat# tlrl-pms	InvivoGen
FSL-1	50 ng/ml	Cat# HY-P2036A	MedChemExpress
Poly(I:C)	100 nM	Cat# B5551	APExBIO
CpG	1 μM	Cat# tlrl-2395-1	InvivoGen
R848	1 μg/ml	Cat# tlrl-r848	InvivoGen
Corticosterone	50 μM	Cat# HY-B1618	MedChemExpress
Dopamine	100 μM	Cat# HY-B0451	MedChemExpress
Norepinephrine	1 μM	Cat# HY-13715	MedChemExpress
Serotonin	10 μM	Cat# HY-B1473A	MedChemExpress
G-CSF	50 ng/ml	Cat# 250-05	PeproTech
M-CSF	10 ng/ml	Cat# 315-02	PeproTech
GM-CSF	200 ng/ml	Cat# 315-03	PeproTech
IFN-γ	50 ng/ml	Cat# 315-05-20	PeproTech
IL-6	50 ng/ml	Cat# 216-16	PeproTech
TNF-α	20 ng/ml	Cat# 210-TA-010	R&D Systems
IL-1β	30 ng/ml	Cat# 201-LB-005	R&D Systems
IL-12	10 ng/ml	Cat# 210-12	PeproTech
IL-23	20 ng/ml	Cat# 1290-IL	R&D Systems
IL-3	10 ng/ml	Cat# 213-13	PeproTech
IL-13	20 ng/ml	Cat# 210-13	PeproTech
TGF-β	2 ng/ml	Cat# 7666-MB	R&D Systems
IL-10	100 ng/ml	Cat# 217-IL-005	R&D Systems

Table S3. Fluorochrome-conjugated antibodies used for flow cytometry

Antigen	Fluorochrome	Identifier	Supplier
Annexin V	FITC	Cat# 556419	BD Pharmingen
B220	Brilliant Violet 421	Clone# RA3-6B2; Cat# 103240	Biolegend
B220	Brilliant Violet 711	Clone# RA3-6B2; Cat# 103255	Biolegend
CD11b	PE-CF594	Clone# M1/70; Cat# 101256	Biolegend
CD11b	Alexa Fluor 700	Clone# M1/70; Cat# 101222	Biolegend
CD11b	PE/Cyanine7	Clone# M1/70; Cat# 552850	BD Pharmingen
CD3	PE	Clone# 17A2; Cat# 100206	Biolegend
CD4	PE-Cyanine7	Clone# GK1.5; Cat# 100422	Biolegend
CD45	Brilliant Violet 570	Clone# 30-F11; Cat# 103136	Biolegend
CD45	Alexa Fluor 700	Clone# 30-F11; Cat# 103128	Biolegend
F4/80	APC	Clone# BM8; Cat# 123116	Biolegend
F4/80	PE/Cyanine7	Clone# BM8; Cat# 123114	Biolegend
Gr-1	FITC	Clone# RB6-8C5; Cat# 11-5931-85	eBioscience
Gr-1	APC	Clone# RB6-8C5; Cat# 108412	Biolegend
Ly6C	APC/Cy7	Clone# HK1.4; Cat# 128026	Biolegend
Ly6G	PE-CF594	Clone# 1A8; Cat# 562700	BD Pharmingen
Ly6G	Brilliant Violet 785	Clone# 1A8; Cat# 127645	Biolegend
Tim-4	PE	Clone# RMT4-54; Cat# 130006	Biolegend

Abbreviations: APC, allophycocyanin; Cy, cyanine; FITC, fluorescein isothiocyanate; PE, phycoerythrin.

Table S4. Primer sequences used for RT-PCR analysis

GENE	Forward primer sequence 5' -3'	Reverse primer sequence 5' -3'
Actb	GGCTGTATTCCCCTCCATCG	CCAGTTGGTAACAATGCCATGT
Chat	CCCCAAAGATGCCTGTA	CCAAACCGCTTCACAAT
Chrna1	CACCCACATCATGCCCGAGT	TGCAAACACAGCCAGCGTCCC
Chrna2	CGGGTGCCCCGGTGGCTGATGA	GAGGTGACAGCAGGATCTCACTAG
Chrna3	GCTATTGCCACCACCGTA	CAGTTCCTAAAATGCACACCA
Chrna4	CTAGCAGCCACATAGAGACCC	CACGCCCATCATAGAACAGGT
Chrna5	TCAACATCCACCACCGCTCT	AAAAGCCCTAGCGTCCCAA
Chrna6	CCACCAGCCATCTTTAAGAGC	TGTAAAACATGGGCAACCTCC
Chrna7	ACACAGTAACCATGCGCCGTA	ACAATCACTGTCACGACCACT
Chrna9	GTCCTCTACAACAAGGCCGAT	TGAACCTCCCATTCCACGTCT
Chrna10	TGGCTCACAAGCTGTTTCGTGACC	CCGCCTTGTTGTAAAGTACGAT
Chrnb1	AACTCATCAGCCTGAACGAGA	AGAGCCCTCGAAAGATACCAC
Chrnb2	CAACTCTATGGCGCTGCT	CGGACTTTCTTCATATTGTCG
Chrnb3	CTGAACATGAAGACGCACTCC	GCTGGATTTCACAATGGCCTT
Chrnb4	CCGCTACAACAACCTGATCCG	AACACGATGTCAGGCAACCAG
Chrnd	AACAACAATGATGGCTCGTTC	ACTATCTCCCACTCACCGTTC
Chrne	CACGGCAGCTTTTACCGAGA	TGGCAACCACCATGACGAA
Chrng	AGGCCCTCACAACTAACGTCT	CTGCAAGTTGATCTCACTGGTGCT
Chrm1	CAAGCAGCCTCCCAAAAGCTC	ACGTAGCAAAGCCAGTAGCC
Chrm2	ATGCACACCAACAAGTACCAC	AGCCAGTAGCCAATTGTCCA
Chrm3	TACCAGGCCACTCACTTACCG	CAGCCAGCTCTTTGGTACGTT
Chrm4	ACAGTGCCTGATAACCAGT	AGGTTTCTTAATGCTCGGCTT
Chrm5	GAAGCACCTCAACAACGGGAA	GGTCCCGTCAGCTTTTACCAC
Erk2	TGAGAACATCATTGGCATCA	CAGCAGGAGGTTGGAAGG
Myd88	TGGTGGTGGTTGTTTCTG	TCTTCATCGCCTTGTATTT
p38	CAAGGTCACTGGAGGAAT	AATTATGTCAGCCGAATGT
Trif	TTGGGGACATACGTTACACTCC	CGGTGTGTTACATAGCTTGCTG

Table S5. The sequences of siRNAs

siRNA	sense sequence 5' -3'	antisense sequence 5' -3'
si-NC	UUCUCCGAACGUGUCACGUTT	ACGUGACACGUUCGGAGAATT
si-*Erk2*-1	GTACAGAGCTCCAGAAATTdTdT	AATTTCTGGAGCTCTGTACdCdA
si-*Erk2*-2	CAAAGAACCCTAAGAGAGAdTdT	TCTCTCTTAGGGTTCTTTGdAdC
si-*Myd88*-1	ACGATTATCTACAGAGCAAdTdT	TTGCTCTGTAGATAATCGTdCdA
si- *Myd88*-2	CAGCGAGCTAATTGAGAAAdTdT	TTTCTCAATTAGCTCGCTGdGdC
si-*p38*-1	GGGCTGAAGTATATACATTdTdT	AATGTATATACTTCAGCCCdTdC
si- *p38-2*	CAAGGTCACTGGAGGAATTdTdT	AATTCCTCCAGTGACCTTGdCdG
si-*Trif*-1	GCATACAGCTGGAGGCAGAdTdT	TCTGCCTCCAGCTGTATGCdTdC
si-*Trif*-2	CCAAGAACCTGAGGAGATAdTdT	TATCTCCTCAGGTTCTTGGdCdA

Table S6. Fluorochrome-conjugated antibodies used for backbone markers stained with PE-conjugated variable antibodies (LegendScreen kit)

Antigen	Fluorochrome	Identifier	Supplier
CD45	PerCP/Cyanine5.5	Clone# 30-F11; Cat# 103132	Biolegend
CD45	Brilliant Violet 570	Clone# 30-F11; Cat# 103136	Biolegend
CD45	Alexa Fluor 700	Clone# 30-F11; Cat# 103128	Biolegend
B220	Brilliant Violet 711	Clone# RA3-6B2; Cat# 103255	Biolegend
CD11b	PE-CF594	Clone# M1/70; Cat# 101256	Biolegend
CD3	PE/Cyanine7	Clone# 17A2; Cat# 100220	Biolegend
F4/80	APC	Clone# BM8; Cat# 123116	Biolegend
Ly6C	Brilliant Violet 510	Clone# HK1.4; Cat# 128033	Biolegend
Ly6G	Brilliant Violet 785	Clone# 1A8; Cat# 127645	Biolegend
MHC-II	Brilliant Violet 421	Clone# M5/114.15.2; Cat# 107632	Biolegend
Zombie	APC/Cy7	Cat# 423101	Biolegend

Abbreviations: APC, allophycocyanin; Cy, cyanine; PE, phycoerythrin.

Dataset S1 (separate file). Summary of marker gene scores for identifying peritoneal lavage cell subtypes using InfinityFlow analysis.

Dataset S2 (separate file). DEG analysis for peritoneal macrophage cluster marker genes in scRNA-seq data.

Dataset S3 (separate file). DEGs between GFP-positive and negative small peritoneal macrophages in scRNA-seq data.

Dataset S4 (separate file). Characteristic gene markers of cell subsets in WT and *Lyz2*-Cre mediated *Chat*-deficient peritoneal lavage from scRNA-seq analysis.

Dataset S5 (separate file). DEGs between WT and *Lyz2*-Cre mediated *Chat*-deficient peritoneal cells in scRNA-seq data.

Dataset S6 (separate file). Transcript abundance measured in TPM for BMDMs treated with various stimuli in bulk RNA-seq data.
